# Associations between cardiometabolic indices and the onset of metabolic dysfunction-associated steatotic liver disease as well as its progression to liver fibrosis: a cohort study

**DOI:** 10.1186/s12933-025-02716-6

**Published:** 2025-04-03

**Authors:** Ziping Song, Xinlei Miao, Shuang Liu, Manling Hu, Xiaoling Xie, Yuting Sun, Song Leng

**Affiliations:** 1 Health Management Center, The Second Hospital of Dalian Medical University, No.467, Zhongshan Road, Dalian, 116023 China; 2 Department of Gastroenterology, The Second Hospital of Dalian Medical University, Dalian, 116023 Liaoning China; 3 School of Public Health, Dalian Medical University, Dalian, 116044 Liaoning China

**Keywords:** Triglyceride-glucose, Cardiometabolic index, Metabolic dysfunction-associated steatotic liver disease, Liver fibrosis, Insulin resistance

## Abstract

**Background:**

This study sought to examine the associations between cardiometabolic indices and the onset of metabolic dysfunction-associated steatotic liver disease (MASLD) as well as its progression to liver fibrosis.

**Methods:**

This study comprised 25,366 subjects aged 18 years and older, free of MASLD at baseline, from the Dalian Health Management Cohort (DHMC). Cardiometabolic indices include cardiometabolic index (CMI), atherogenic index of plasma (AIP), triglyceride glucose (TyG), triglyceride glucose-body mass index (TyG-BMI), triglyceride glucose-waist circumference (TyG-WC) and triglyceride glucose-waist height ratio (TyG-WHtR). All participants were categorized into quartile groups based on cardiometabolic indices. Cox proportional hazards regression models and restricted cubic splines were employed to examine the relationship between cardiometabolic indices and the incidence of MASLD as well as its progression to liver fibrosis, and analyses were performed between different subgroups. Mediation analysis was employed to explore how obesity and inflammation serve as mediators in the connection between cardiometabolic indices and MASLD. To evaluate the predictive ability of cardiometabolic indices for the onset of MASLD, the time-dependent receiver operating characteristic (ROC) curve was utilized.

**Results:**

A total of 5378 (21.2%) individuals developed MASLD during the follow-up period of 82,445 person-years. Multivariates Cox regression analyses showed that participants in the highest quartile of cardiometabolic indices had greater risk of MASLD than those in the lowest quartile (CMI: HR = 6.11, 95% CI 5.45–6.86; AIP: HR = 4.58, 95% CI 4.11–5.10; TyG: HR = 3.55, 95% CI 3.21–3.92; TyG-BMI: HR = 13.55, 95% CI 11.80–15.57; TyG-WC: HR = 12.52, 95% CI 10.93–14.34; TyG-WHtR: HR = 11.37, 95% CI 9.96–12.98). TyG-BMI (HR = 1.36, 95% CI 1.18–1.57), but not other cardiometabolic indices, was associated with liver fibrosis. Mediation analysis indicated that BMI mediated 40.4%, 33.2%, 36.5%, − 10.4%, 37.4%, 48.5% of the associations between CMI, AIP, TyG, TyG-BMI, TyG-WC, TyG-WHtR and MASLD. Time-dependent ROC curves demonstrated that TyG-BMI had a superior predictive ability for MASLD onset compared to other indicators.

**Conclusions:**

The risk of developing MASLD increases as the level of cardiometabolic indices increases. Obesity may serve as a mediating factor in the aforementioned association. TyG-BMI showed the strongest association with the onset of MASLD and its progression to liver fibrosis, proved to be outperformed other cardiometabolic indicators, and could be the best clinical non-invasive biomarker for early screening of MASLD and liver fibrosis.

**Supplementary Information:**

The online version contains supplementary material available at 10.1186/s12933-025-02716-6.

## Introduction

Metabolic dysfunction-associated steatotic liver disease (MASLD) is characterized by the presence of one or more cardiometabolic criterias, coupled with an absence of other causes of steatosis, which distinguishes it from other forms of steatotic liver disease [1, 2]. With a global uptick in metabolic disorders, the rates of MASLD are similarly on the rise [3]. As the most prevalent chronic non-communicable liver disease worldwide, MASLD presents a significant public health challenge that warrants urgent attention [4]. Research indicates that MASLD not only worsens liver conditions but also has implications for other serious health issues, including kidney diseases, cardiovascular problems, and extrahepatic cancers, leading to substantial social and economic impacts [5–8]. To make matters worse, there is currently no specific treatment for MASLD [9]. Previous studies have shown that patients with compensated disease demonstrate a median survival of approximately 12 years, whereas those diagnosed at the decompensated stage experience a shorter survival of 2–4 years [10]. Intervention and management of unhealthy lifestyle behaviors can effectively improve early hepatic steatosis [9]. Therefore, exploring potential risk factors and convenient measurable biomarkers for MASLD and liver fibrosis is significant for clinical practice.

Epidemiological evidence showed that insulin resistance, inflammation and metabolic factors significantly contribute to the pathophysiology of MASLD [11, 12]. Various cardiometabolic indices such as cardiometabolic index (CMI), atherogenic index of plasma (AIP), triglyceride glucose (TyG), triglyceride glucose-body mass index (TyG-BMI), triglyceride glucose-waist circumference (TyG-WC), triglyceride glucose-waist height ratio (TyG-WHtR), have emerged as significant biomarkers for assessing insulin resistance [13–17]. Although these indicators have been illustrated to be associated with multiple diseases, the specific relationship with the incidence of MASLD and its progression to liver fibrosis has not been fully elucidated [18–22]. In addition, the relative effects of each cardiometabolic related indices are not the same.

Therefore, we sought to investigate the associations between cardiometabolic indices and MASLD as well as its progression to liver fibrosis, and to ascertain the best indicator among them. Furthermore, we planned to explore which factors mediated these relationships.

## Methods

### Study population

This study utilized data from the Dalian Health Management Cohort (DHMC) (ChiCTR2300073363), a large-scale and ongoing study initiated in 2014 based on a physical examination population from the medical examination center of a third-class hospital in Dalian. DHMC aims to investigate the health of the population, deeply explore the risk factors affecting the health of the population, and provide scientific guidance for effectively identifying high-risk and susceptible groups of various chronic diseases and scientifically formulating targeted intervention and prevention strategies. Details of DHMC have been previously reported [23].

This study included 68,994 participants aged 18 and older who had undergone at least two health check-ups between 2014 and 2023. Participants with MASLD at baseline, without data to calculate cardiometabolic indices, with missing covariates, and with other liver diseases and malignancy were excluded. Ultimately, 25,366 individuals were included in this study (Fig. S1). The first visit was defined as baseline, and follow-up to the date of the last visit or the onset of MASLD, whichever came first. We excluded 2721 individuals who lost follow-up and 1011 individuals without required data from 5378 MASLDs. In total, 1646 subjects diagnosed with MASLD were examined to evaluate the relationship between cardiometabolic indices and subsequent liver fibrosis. This study adhered to the Declaration of Helsinki and was approved by the Ethics Review Committee of the Second Hospital of Dalian Medical University (approval number: 2022064). All participants provided informed consent.

### Measurement and definition of cardiometabolic indices

The height and weight of individuals who are barefoot and wear light clothing were measured by a measuring instrument. Waist circumference was measured by professional nurses at the midway point between the lower edge of the ribs and the iliac crest. Fasting venous blood samples were obtained in accordance with established protocols, allowing for the measurement of various parameters including white blood cell (WBC) count, lymphocyte, neutrophil, red blood cell (RBC), platelet, high-density lipoprotein cholesterol (HDL-C), total cholesterol (TC), low-density lipoprotein cholesterol (LDL-C), triglyceride (TG), fasting plasma glucose (FPG), hemoglobin A1c (HbA1c), gamma-glutamyl transferase (GGT), aspartate aminotransferase (AST), alanine aminotransferase (ALT), serum creatinine (Scr) and serum uric acid (SUA) as analyzed by the laboratory. BMI was calculated using the formula weight (kg) divided by height squared (m^2^), while WHtR was derived by dividing waist circumference by height. The calculation formulas of cardiometabolic indices were provided in the Supplementary Materials [13, 18, 24].

### Definition of MASLD and liver fibrosis

Liver ultrasonography was conducted by professional sonographers who were both trained and unaware of the specifics of this study. Fatty liver was diagnosed based on the following features shown in the liver ultrasound: enhanced hepatic parenchymal echogenicity (“bright liver”), far-field echo attenuation, and poor visualization of intrahepatic ductal structures [25]. All participants underwent ultrasound scans by trained sonographers using a standardized protocol. Subsequently re-evaluated by a second certified sonographer. MASLD was defined as steatotic liver disease (SLD) observed via ultrasound and the presence of one or more of the following abnormalities after excluding excessive alcoholic consumption (≥ 140 g/wk for females and ≥ 210 g/wk for males) and other liver diseases: (1) WC ≥ 90/80 cm (for males/females) or BMI ≥ 23 kg/m^2^; (2) HbA1c ≥ 5.7% or FPG ≥ 5.6 mmol/L or type 2 diabetes mellitus or glucose-lowering drug use; (3) Systolic blood pressure (SBP) ≥ 130 mmHg, diastolic blood pressure (DBP) ≥ 85 mmHg or specific drug treatment; (4) TG ≥ 1.7 mmol/L or lipid-lowering treatment; (5) HDL-C < 1.3 mmol/L for females and < 1.0 mmol/L for males or lipid-lowering treatment [2]. Among participants with MASLD, we used NAFLD fibrosis score (NFS) to evaluate the probability of liver fibrosis. NFS = − 1.675 + (0.037 × age in years) + (0.094 × BMI in kg/m^2^) + (1.13 × presence of impaired fasting glucose/diabetes, yes = 1, no = 0) + (0.99 × AST/ALT ratio) − (0.013 × platelet count in × 10^9^/L) − (0.66 × albumin level in g/dL) [26]. The higher probability of liver fibrosis referred to NFS ≥ − 1.455 [27]. The threshold for NFS in people aged 65 and above was 0.12 [28].

### Covariates

Participants’ sex, age, medical history, medication history, smoking and drinking history were obtained through electronic information system. Smoking was categorized as either “never smoke” or “current/former” [29]. Alcohol consumption was defined as drinking at least once a week in the past year and further classified as excessively (≥ 210 g/wk for males and 140 g/wk for females), occasionally (0–210 g/wk for males and 0–140 g/wk for females), or never [29, 30]. After resting for at least 5 min, using an electronic sphygmomanometer measured the subject’s blood pressure. Individuals were classified as hypertensive if they had a prior diagnosis of hypertension, were on antihypertensive medication, or SBP ≥ 140 mmHg or DBP ≥ 90 mmHg [31]. Similarly, those were deemed to have diabetes if they had a history of the condition, were using hypoglycemic drugs or insulin, or presented with a fasting plasma glucose level of 7.0 mmol/L or higher, or a hemoglobin A1c level of 6.5% or above [32]. Dyslipidemia was identified based on any of the following criteria: TG ≥ 200 mg/dL or TC ≥ 240 mg/dL or LDL-C ≥ 160 mg/dL or HDL-C ≤ 40 mg/dL or a history of dyslipidemia, and taking lipid-lowering agents [33].

### Statistical analysis

Continuous variables conforming to non-normal distributions were described as medians and interquartile ranges, and normal distribution were described as means ± standard deviations. One-way ANOVA and the Kruskal–Wallis H test were used to assess differences among the groups, respectively. Data were described as numbers (percentages) for categorical variables and differences were compared using the chi-square test. Missing values of covariates were shown in Fig. S2. Multiple imputation was performed on the missing data using “miceforest”.

The cumulative incidence of MASLD events was assessed by Kaplan–Meier method. The dose–response relationships were examined through restricted cubic spline (RCS). Cox proportional hazards regression models were used to evaluate the association of cardiometabolic indices with MASLD and its components as well as its progression to liver fibrosis. Model 1 was not adjusted. Model 2 adjusted for sex and age. On the basis of model 2, smoking status, drinking status, hypertension, diabetes mellitus, and dyslipidemia were adjusted in model 3. Based on clinical experience and summarized from previous literature, these facctors were identified as confounders by a directed acyclic graph (Fig. S3). Subgroups were created based on age, sex, BMI, hypertensive status, diabetic status, and dyslipidemia status. Mediator effect analyses were used to explore whether the correlation between cardiometabolic indices and MASLD could be explained by obesity (WC, BMI) and inflammation (leukocytes, neutrophils, lymphocytes). Time-dependent receiver operating characteristic (ROC) curves and time-dependent Harrell’s concordance index (C-index) were constructed to estimate predictive ability of cardiometabolic indices for predicting the onset of MASLD. In addition, to ensure the findings were robust, we conducted multiple sensitivity analyses. First, patients who experienced MASLD during the first year of follow-up were excluded to address potential reverse causality. Second, patients taking antihypertensive, hypoglycemic, and lipid-lowing drugs were excluded. Third, the optimal cardiometabolic indices cutoff point was obtained by maximally selected rank statistics facilitated by the ‘maxstat’ package, which divided participants into low and high groups. Fourth, propensity score matching (PSM) was used to assess again. Fifth, we used Metabolic Dysfunction-Associated Fibrosis 5 (MAF-5) to evaluate the probability of liver fibrosis. MAF-5 = − 11.3674 + WC × 0.0282 − BMI × 0.1761 + WC × BMI × 0.0019 + 2.0762 for diabetes + ln (AST) × 2.9207—platelets × 0.0059 [34]. The higher probability of fibrosis referred to MAF-5 ≥ 1 [34].

All statistical analyses were performed by R 4.2.2 and SPSS 27.0, and *P* < 0.05 (two-sided test) was defined as statistical significance.

## Results

### Characteristics of participants

Among 25,366 participants, 11,654 (45.9%) were male. After 82,445 person-years of follow-up, 5378 (21.2%) were diagnosed with MASLD. Compared with those without MASLD, participants with MASLD were more likely to be male, older, had higher levels of WC, BMI, DBP, SBP, WBC, neutrophils, lymphocytes, RBC, hemoglobin, platelet, FPG, TC, LDL-C, TG, AST, ALT, GGT, SUA, Scr, lower HDL-C, and had a higher proportions of smoking, drinking, hypertension, diabetes, dyslipidemia (Table 1).

**Table 1:** Baseline characteristics of the study population with and without MASLD

Variables	Overall (n = 25,366)	Non-MASLD (n = 19,988)	MASLD (n = 5378)	*P* value
Sex, male, n (%)	11,654 (45.94)	8209 (41.07)	3445 (64.06)	< 0.001
Age, years	40.00 (31.00, 51.00)	39.00 (31.00, 50.00)	43.00 (33.00, 52.00)	< 0.001
Smoking status, n (%)	644 (2.54)	439 (2.20)	205 (3.81)	< 0.001
Drinking status, n (%)	786 (3.10)	583 (2.92)	203 (3.77)	< 0.001
BMI, kg/m^2^	23.14 ± 2.96	22.62 ± 2.81	25.08 ± 2.69	< 0.001
WC, cm	80.80 ± 9.52	79.23 ± 9.21	86.60 ± 8.32	< 0.001
SBP, mmHg	124.38 ± 16.57	123.29 ± 16.49	128.43 ± 16.23	< 0.001
DBP, mmHg	74.80 ± 10.57	74.04 ± 10.33	77.62 ± 10.97	< 0.001
WBC, 10^9^/L	5.93 ± 1.45	5.82 ± 1.41	6.31 ± 1.52	< 0.001
Neutrophils, 10^9^/L	3.47 ± 1.13	3.41 ± 1.11	3.71 ± 1.18	< 0.001
Lymphocytes, 10^9^/L	1.98 ± 0.53	1.95 ± 0.52	2.10 ± 0.56	< 0.001
RBC, 10^12^/L	4.76 ± 0.44	4.71 ± 0.43	4.92 ± 0.43	< 0.001
Hemoglobin, g/L	142.05 ± 15.85	140.54 ± 15.69	147.67 ± 15.16	< 0.001
Platelet, 10^9^/L	238.28 ± 53.06	237.84 ± 52.85	239.91 ± 53.81	< 0.001
FPG, mmol/L	5.37 (5.10, 5.70)	5.34 (5.07, 5.65)	5.50 (5.20, 5.85)	< 0.001
TC, mmol/L	4.81 ± 0.88	4.78 ± 0.87	4.92 ± 0.90	< 0.001
TG, mmol/L	1.15 (0.84, 1.59)	1.08 (0.80, 1.48)	1.44 (1.07, 2.00)	< 0.001
HDL-C, mmol/L	1.41 ± 0.32	1.45 ± 0.32	1.26 ± 0.27	< 0.001
LDL-C, mmol/L	2.57 ± 0.69	2.53 ± 0.68	2.70 ± 0.68	< 0.001
ALT, U/L	16.48 (12.40, 22.92)	15.69 (12.00, 21.27)	20.15 (15.23, 28.00)	< 0.001
AST,U/L	18.89 (16.00, 22.04)	18.53 (16.00, 21.97)	19.81 (17.00, 23.44)	< 0.001
GGT, U/L	14.69 (10.86, 22.00)	13.59 (10.16, 19.86)	19.66 (14.00, 30.00)	< 0.001
SUA, μmol/L	326.07 ± 84.28	316.00 ± 81.08	363.55 ± 85.39	< 0.001
Scr, μmol/L	63.56 (54.43, 75.92)	62.10 (53.95, 74.63)	69.00 (57.64, 79.01)	< 0.001
CMI	0.40 (0.26, 0.62)	0.36 (0.24, 0.55)	0.60 (0.40, 0.89)	< 0.001
AIP	− 0.07 ± 0.25	− 0.11 ± 0.24	0.07 ± 0.25	< 0.001
TyG	8.54 ± 0.51	8.47 ± 0.48	8.79 ± 0.51	< 0.001
TyG-BMI	198.12 ± 31.30	192.03 ± 29.15	220.77 ± 28.47	< 0.001
TyG-WC	691.85 ± 104.18	672.86 ± 98.56	762.44 ± 93.59	< 0.001
TyG-WHtR	4.12 ± 0.58	4.02 ± 0.55	4.49 ± 0.52	< 0.001
Hypertension, n (%)	4542 (17.91)	3183 (15.92)	1359 (25.27)	< 0.001
Diabetes, n (%)	1009 (3.98)	672 (3.36)	337 (6.27)	< 0.001
Dyslipidemia, n (%)	4789 (18.88)	3025 (15.13)	1764 (32.80)	< 0.001

The data are presented as the means ± SDs, n (%), or medians (quartile 1, quartile 3)

MASLD metabolic dysfunction-associated steatotic liver disease, BMI body mass index, WC waist circumference, SBP systolic blood pressure, DBP diastolic blood pressure, WBC white blood cell, RBC red blood cell, FPG fasting plasma glucose, TC total cholesterol, TG triglyceride, HDL-C high-density lipoprotein cholesterol, LDL-C low-density lipoprotein cholesterol, ALT alanine aminotransferase, AST aspartate aminotransferase, GGT gamma-glutamyl transpeptidase, SUA serum uric acid, Scr serum creatinine, CMI cardiometabolic index, AIP atherogenic index of plasma, TyG triglyceride-glucose index, TyG-BMI triglyceride-glucose × body mass index, TyG-WC triglyceride-glucose × waist circumference, TyG-WHtR triglyceride-glucose × waist circumference/height

Baseline characteristics of participants categorized by quartiles of cardiometabolic indices are also shown in Tables S1–S6. Individuals exhibiting elevated levels of cardiometabolic indices were predominantly male, older, and demonstrated increased levels of metabolic related factors.

### The incidence density of MASLD

The incidence density of MASLD was 65.23 per 1000 person-years. In CMI quartile groups were 17.25, 41.45, 76.62 and 144.97 per 1000 person-years, respectively. In AIP quartile groups were 20.50, 45.27, 73.98 and 136.50 per 1000 person-years, respectively. In TyG quartile groups were 24.85, 48.27, 74.59 and 128.13 per 1000 person-years, respectively. In TyG-BMI quartile groups were 10.16, 36.41, 79.24 and 164.14 per 1000 person-years, respectively. In TyG-WC quartile groups were 11.86, 40.28, 78.60 and 159.79 per 1000 person-years, respectively. In TyG-WHtR quartile groups were 11.92, 43.25, 80.65 and 150.89 per 1000 person-years, respectively. The analysis by age group found that the incidence density of MASLD was the highest in subjects aged 45–59 years, followed by age 60 and over and the lowest was aged 18–44 years (Table S7).

### Association between cardiometabolic indices and the incidence of MASLD as well as components

**Fig. 1:**
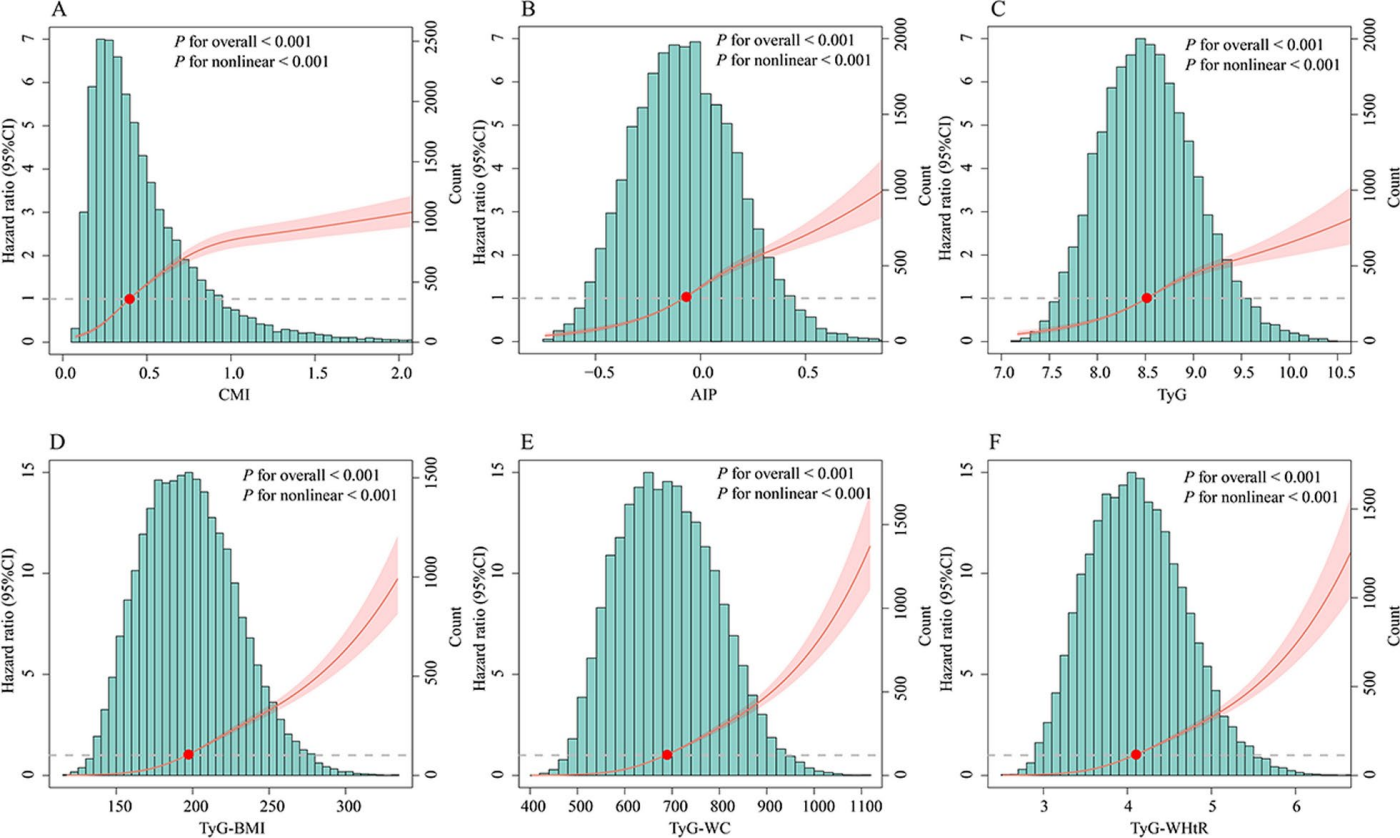
Restricted cubic spline analysis of cardiometabolic biomarkers with MASLD. CMI cardiometabolic index, AIP atherogenic index of plasma, TyG triglyceride-glucose index, TyG-BMI triglyceride-glucose × body mass index, TyG-WC triglyceride-glucose × waist circumference, TyG-WHtR triglyceride-glucose × waist circumference/height, MASLD metabolic dysfunction-associated steatotic liver disease

Kaplan–Meier curves showed that individuals in the higher cardiometabolic indices quartiles had greater cumulative incidence of MASLD than those in the lowest quartiles (Fig. S4). The RCS curves indicated that cardiometabolic indices and MASLD exhibited nonlinear associations (*P* for overall < 0.001, *P* for nonlinear < 0.001) (Fig. 1). Compared with individuals in the lowest quartiles, the adjusted HR (95%CI) in the highest quartiles of CMI, AIP, TyG, TyG-BMI, TyG-WC and TyG-WHtR were 6.11 (5.45–6.86), *P* < 0.001; 4.58 (4.11–5.10), *P* < 0.001; 3.55 (3.21–3.92), *P* < 0.001; 13.55 (11.80–15.57), *P* < 0.001; 12.52 (10.93–14.34), *P* < 0.001; 11.37 (9.96–12.98), *P* < 0.001, respectively (Table 2). The cardiometabolic indices were also associated with MASLD components, including obesity, raised FPG or DM, raised BP, raised TG and reduced HDL-C (Table S8).

**Table 2:** Associations between cardiometabolic indices and the incidence of MASLD

Variables	Model 1 *HR* (95% *CI*)	*P* value	Model 2 *HR* (95% *CI*)	*P* value	Model 3 *HR* (95% *CI*)	*P* value
CMI						
Per SD increase	1.41 (1.39, 1.42)	< 0.001	1.34 (1.32, 1.36)	< 0.001	1.28 (1.26, 1.30)	< 0.001
Quartiles						
Q1	Reference		Reference		Reference	
Q2	2.46 (2.18, 2.76)	< 0.001	2.24 (1.99, 2.52)	< 0.001	2.23 (1.98, 2.51)	< 0.001
Q3	4.62 (4.14, 5.16)	< 0.001	3.98 (3.56, 4.45)	< 0.001	3.87 (3.46, 4.33)	< 0.001
Q4	8.89 (7.99, 9.88)	< 0.001	6.96 (6.23, 7.77)	< 0.001	6.11 (5.45, 6.86)	< 0.001
*P*_trend_		< 0.001		< 0.001		< 0.001
AIP						
Per SD increase	1.88 (1.83, 1.92)	< 0.001	1.72 (1.68, 1.77)	< 0.001	1.68 (1.63, 1.74)	< 0.001
Quartiles						
Q1	Reference		Reference		Reference	
Q2	2.26 (2.03, 2.52)	< 0.001	2.05 (1.83, 2.29)	< 0.001	2.03 (1.82, 2.27)	< 0.001
Q3	3.75 (3.38, 4.16)	< 0.001	3.17 (2.85, 3.52)	< 0.001	3.06 (2.75, 3.40)	< 0.001
Q4	7.01 (6.35, 7.74)	< 0.001	5.36 (4.84, 5.94)	< 0.001	4.58 (4.11, 5.10)	< 0.001
*P*_trend_		< 0.001		< 0.001		< 0.001
TyG						
Per SD increase	1.78 (1.74, 1.83)	< 0.001	1.65 (1.60, 1.69)	< 0.001	1.56 (1.51, 1.61)	< 0.001
Quartiles						
Q1	Reference		Reference		Reference	
Q2	1.99 (1.80, 2.21)	< 0.001	1.82 (1.64, 2.02)	< 0.001	1.78 (1.61, 1.97)	< 0.001
Q3	3.14 (2.85, 3.46)	< 0.001	2.66 (2.41, 2.94)	< 0.001	2.53 (2.29, 2.79)	< 0.001
Q4	5.50 (5.02, 6.03)	< 0.001	4.33 (3.93, 4.76)	< 0.001	3.55 (3.21, 3.92)	< 0.001
* P*_trend_		< 0.001		< 0.001		< 0.001
TyG-BMI						
Per SD increase	2.27 (2.21, 2.33)	< 0.001	2.19 (2.13, 2.25)	< 0.001	2.13 (2.07, 2.19)	< 0.001
Quartiles						
Q1	Reference		Reference		Reference	
Q2	3.62 (3.13, 4.18)	< 0.001	3.50 (3.03, 4.04)	< 0.001	3.46 (2.99, 4.00)	< 0.001
Q3	7.98 (6.97, 9.14)	< 0.001	7.27 (6.33, 8.35)	< 0.001	6.99 (6.09, 8.03)	< 0.001
Q4	17.04 (14.93, 19.45)	< 0.001	15.09 (13.16, 17.30)	< 0.001	13.55 (11.80, 15.57)	< 0.001
* P*_trend_		< 0.001		< 0.001		< 0.001
TyG-WC						
Per SD increase	2.29 (2.23, 2.35)	< 0.001	2.33 (2.26, 2.40)	< 0.001	2.26 (2.18, 2.34)	< 0.001
Quartiles						
Q1	Reference		Reference		Reference	
Q2	3.45 (3.02, 3.94)	< 0.001	3.48 (3.04, 3.98)	< 0.001	3.43 (3.00, 3.93)	< 0.001
Q3	6.84 (6.02, 7.76)	< 0.001	6.87 (6.02, 7.83)	< 0.001	6.49 (5.68, 7.40)	< 0.001
Q4	14.30 (12.65, 16.17)	< 0.001	14.48 (12.67, 16.54)	< 0.001	12.52 (10.93, 14.34)	< 0.001
* P*_trend_		< 0.001		< 0.001		< 0.001
TyG-WHtR						
Per SD increase	2.11 (2.06, 2.16)	< 0.001	2.22 (2.16, 2.29)	< 0.001	2.16 (2.09, 2.23)	< 0.001
Quartiles						
Q1	Reference		Reference		Reference	
Q2	3.65 (3.20, 4.17)	< 0.001	3.55 (3.11, 4.06)	< 0.001	3.50 (3.06, 4.01)	< 0.001
Q3	6.96 (6.13, 7.90)	< 0.001	6.63 (5.83, 7.55)	< 0.001	6.34 (5.57, 7.22)	< 0.001
Q4	13.32 (11.77, 15.08)	< 0.001	13.00 (11.42, 14.80)	< 0.001	11.37 (9.96, 12.98)	< 0.001
* P*_trend_		< 0.001		< 0.001		< 0.001

Model 1: Unadjusted

Model 2: Adjusted for age and sex

Model 3: Adjusted for age, sex, smoking status, drinking status, hypertension, diabetes and dyslipidemia

*HR* Hazard ratio, *CI* Confidence interval, *MASLD* Metabolic dysfunction-associated steatotic liver disease, *CMI* Cardiometabolic index, *AIP* Atherogenic index of plasma, *TyG* Triglyceride-glucose index, *TyG-BMI* Triglyceride-glucose × body mass index, *TyG-WC* Triglyceride-glucose × waist circumference, *TyG-WHtR* Triglyceride-glucose × waist circumference/height

### Association between cardiometabolic indices and the incidence of liver fibrosis

Using NFS and MAF-5, 256 and 147 MASLD patients were respectively identified with a higher probability of liver fibrosis. Compared with participants without liver fibrosis, participants with fibrosis tended to be male, older, had higher levels of BMI, WC, SBP, DBP, hemoglobin, FPG, GGT, SUA and had a higher proportions of smoking, drinking, hypertension, diabetes (Table S9). A linear correlation was observed between CMI, AIP, TyG-WC and liver fibrosis. TyG, TyG-BMI and TyG-WHtR exhibited nonlinear correlations with liver fibrosis (Fig. S5). Compared with the lowest quartile of TyG-BMI, patients in the highest quartiles had 1.75 times (HR 1.75, 95% CI 1.21–2.54, *P* = 0.003) and 4.47 times (HR 4.47, 95% CI 2.40–8.32, *P* < 0.001) higher risks of developing higher NFS and MAF-5, respectively (Table 3, Table S10).

**Table 3:** Association between cardiometabolic indices and non-invasive fibrosis score

Variables	Model 1 *HR* (95% *CI*)	*P* value	Model 2 *HR* (95% *CI*)	*P* value	Model 3 *HR* (95% *CI*)	*P* value
MASLD with NFS ≥ -1.455(0.12 for ≥ 65 years)						
CMI						
Per SD increase	0.98 (0.88, 1.10)	0.781	0.97 (0.83, 1.13)	0.718	0.94 (0.70, 1.26)	0.676
Quartiles						
Q1	Reference		Reference		Reference	
Q2	0.95 (0.66, 1.35)	0.761	0.87 (0.61, 1.25)	0.465	1.00 (0.70, 1.44)	0.982
Q3	0.99 (0.70, 1.41)	0.960	0.96 (0.67, 1.36)	0.814	0.88 (0.61, 1.26)	0.473
Q4	1.10 (0.78, 1.55)	0.595	0.97 (0.69, 1.37)	0.855	0.89 (0.59, 1.34)	0.571
*P*_trend_		0.543		0.982		0.451
AIP						
Per SD increase	0.98 (0.86, 1.10)	0.694	0.96 (0.85, 1.09)	0.568	0.90 (0.78, 1.04)	0.147
Quartiles						
Q1	Reference		Reference		Reference	
Q2	0.87 (0.60, 1.25)	0.447	0.87 (0.60, 1.26)	0.451	1.05 (0.72, 1.53)	0.800
Q3	0.89 (0.64, 1.24)	0.497	0.88 (0.63, 1.22)	0.435	0.83 (0.59, 1.16)	0.272
Q4	1.01 (0.73, 1.39)	0.958	0.99 (0.72, 1.36)	0.938	0.90 (0.59, 1.39)	0.635
*P*_trend_		0.993		0.885		0.342
TyG						
Per SD increase	1.45 (1.29, 1.63)	< 0.001	1.28 (1.13, 1.44)	< 0.001	1.03 (0.87, 1.21)	0.748
Quartiles						
Q1	Reference		Reference		Reference	
Q2	0.88 (0.58, 1.34)	0.555	0.87 (0.57, 1.33)	0.527	0.65 (0.42, 1.00)	0.050
Q3	1.59 (1.10, 2.31)	0.014	1.21 (0.84, 1.76)	0.310	0.86 (0.58, 1.28)	0.460
Q4	2.20 (1.55, 3.13)	< 0.001	1.65 (1.16, 2.36)	0.005	0.97 (0.61, 1.55)	0.898
*P*_trend_		< 0.001		0.001		0.709
TyG-BMI						
Per SD increase	1.47 (1.29, 1.67)	< 0.001	1.52 (1.33, 1.74)	< 0.001	1.36 (1.18, 1.57)	< 0.001
Quartiles						
Q1	Reference		Reference		Reference	
Q2	1.00 (0.67, 1.51)	0.988	0.98 (0.65, 1.48)	0.933	1.05 (0.69, 1.59)	0.827
Q3	1.34 (0.91, 1.96)	0.136	1.09 (0.74, 1.60)	0.664	0.97 (0.65, 1.45)	0.896
Q4	2.30 (1.62, 3.27)	< 0.001	2.20 (1.55, 3.13)	< 0.001	1.75 (1.21, 2.54)	0.003
*P*_trend_		< 0.001		< 0.001		0.002
TyG-WC						
Per SD increase	2.56 (1.99, 3.27)	< 0.001	1.94 (1.48, 2.56)	< 0.001	1.47 (1.08, 2.00)	0.013
Quartiles						
Q1	Reference		Reference		Reference	
Q2	1.31 (0.86, 1.99)	0.206	1.03 (0.67, 1.58)	0.881	1.01 (0.66, 1.55)	0.958
Q3	1.67 (1.12, 2.50)	0.012	1.21 (0.80, 1.83)	0.366	1.18 (0.77, 1.79)	0.450
Q4	2.96 (2.05, 4.28)	< 0.001	1.84 (1.23, 2.74)	0.003	1.43 (0.93, 2.21)	0.106
*P*_trend_		< 0.001		< 0.001		0.056
TyG-WHtR						
Per SD increase	2.17 (1.83, 2.58)	< 0.001	1.56 (1.30, 1.87)	< 0.001	1.30 (1.06, 1.59)	0.012
Quartiles						
Q1	Reference		Reference		Reference	
Q2	1.06 (0.68, 1.66)	0.784	0.88 (0.56, 1.38)	0.580	0.87 (0.56, 1.36)	0.550
Q3	1.85 (1.25, 2.75)	0.002	1.25 (0.84, 1.87)	0.267	1.09 (0.72, 1.66)	0.678
Q4	3.33 (2.31, 4.80)	< 0.001	1.89 (1.29, 2.76)	0.001	1.44 (0.95, 2.19)	0.086
*P*_trend_		< 0.001		< 0.001		0.027

Model 1: Unadjusted

Model 2: Adjusted for age and sex

Model 3: Adjusted for age, sex, smoking status, drinking status, hypertension, diabetes and dyslipidemia

*HR* Hazard ratio, *CI* Confidence interval, *MASLD* Metabolic dysfunction-associated steatotic liver disease, *CMI* Cardiometabolic index, *AIP* Atherogenic index of plasma, *TyG* Triglyceride-glucose index, *TyG-BMI* Triglyceride-glucose × body mass index, *TyG-WC* Triglyceride-glucose × waist circumference, *TyG-WHtR* Triglyceride-glucose × waist circumference/height

### Subgroup and sensitivity analyses between cardiometabolic indices and MASLD

Subgroup analyses revealed that in each subgroup stratified, the positive associations between cardiometabolic indices and MASLD were consistent, but significant associations were more likely to be observed among individuals who were females, and individuals without obesity, without hypertension (Tables S11–S16). In the sensitivity analyses, excluding individuals with MASLD occurring within first year of follow-up (n = 1107) and excluding individuals who used antihypertensive, hypoglycemic (n = 1518), the results were similarly (Tables S17–S18). After dividing participants into low and high groups according to the optimal cutoff point, we found that compared with the low group, individuals in the high group had a 2.78 times, 2.22 times, 2.01 times, 3.60 times, 3.25 times, 3.27 times greater risk of developing MASLD, respectively (CMI: HR = 2.78, 95% CI 2.60–2.97; AIP: HR = 2.22, 95% CI 2.09–2.36; TyG: HR = 2.01, 95% CI 1.89–2.14; TyG-BMI: HR = 3.60, 95% CI 3.38–3.84; TyG-WC: HR = 3.25, 95% CI 3.03–3.47; TyG-WHtR: HR = 3.27, 95% CI 3.06–3.49) (Table S19). In addition, after PSM, the results still indicated a positive association between cardiometabolic indices and MASLD (Figs. S6–S11, Table S20).

### Mediation analyses between cardiometabolic indices and MASLD

**Fig. 2:**
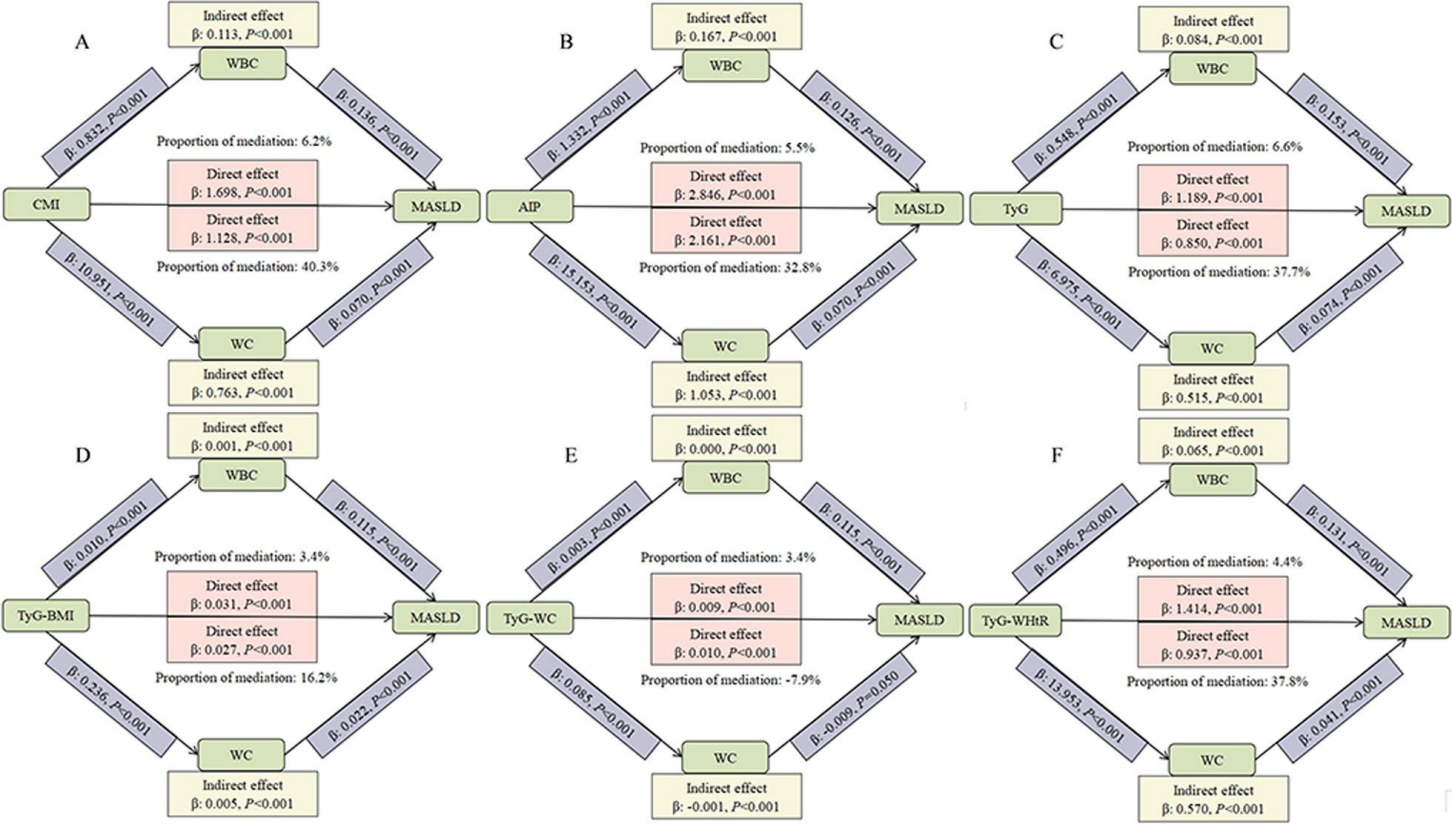
Mediation analysis of cardiometabolic indices with MASLD. CMI cardiometabolic index, AIP atherogenic index of plasma, TyG triglyceride-glucose index, TyG-BMI triglyceride-glucose × body mass index, TyG-WC triglyceride-glucose × waist circumference, TyG-WHtR triglyceride-glucose × waist circumference/height, MASLD metabolic dysfunction-associated steatotic liver disease, WBC white blood cell, WC waist circumference

Mediation analyses showed that WC and BMI mediated a more significant proportion of indirect effects in the associations between cardiometabolic indices and MASLD (Fig. 2, Table S21). For CMI, AIP, TyG and its derived indicators, the mediation proportions of WC-mediated MASLD were 40.3%, 32.8%, 37.7%, 16.2%, -7.9%, 37.8%, respectively. BMI mediated 40.4%, 33.2%, 36.5%, − 10.4%, 37.4%, 48.5% of the associations between CMI, AIP, TyG and its derived indicators and MASLD. Figure 2 showed the proportion of indirect effects mediated by WBC.

### Predicive performance comparison

**Fig. 3:**
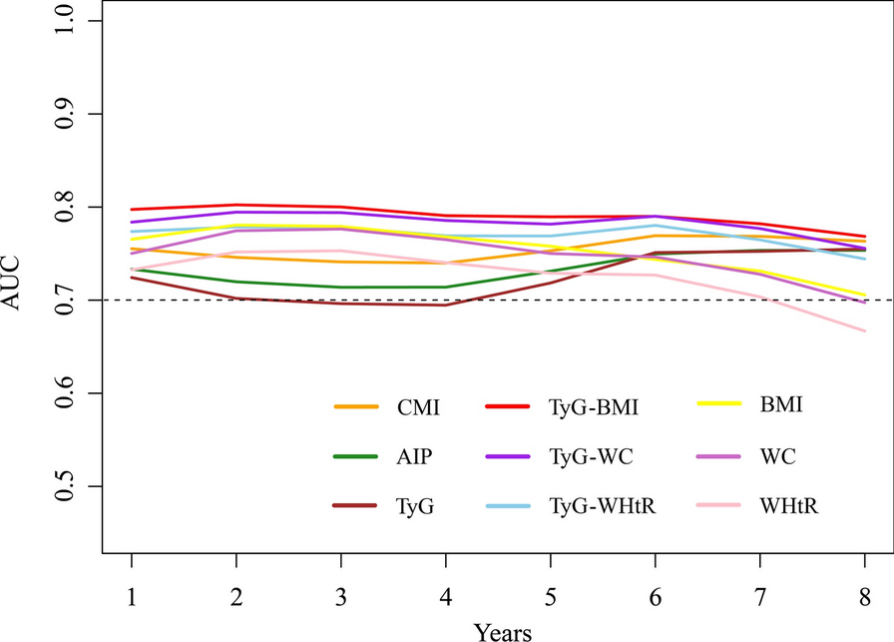
Time-dependent predictive performance of cardiometabolic indices for predicting the onset of MASLD

Time-dependent ROC curves showed that TyG-BMI had the highest AUC in predicting the onset of MASLD (Figs. 3, and S12). To further validate the predictive performance of cardiometabolic indices for MASLD onset, we computed time-dependent Harrell’s concordance indices (C-index). Among all indices, TyG-BMI demonstrated the highest predictive ability (C-index = 0.768, 95% CI 0.762–0.774), followed by TyG-WC (C-index = 0.758, 95% CI 0.752–0.764) and TyG-WHtR (C-index = 0.745, 95% CI 0.739–0.751). Conventional anthropometric measures such as BMI (C-index = 0.744, 95% CI 0.738–0.750), WC (C-index = 0.735, 95% CI 0.729–0.741), and WHtR (C-index = 0.716, 95% CI 0.710–0.722) exhibited moderate predictive value, while CMI (C-index = 0.725, 95% CI 0.717–0.733) and AIP (C-index = 0.703, 95% CI 0.695–0.711) showed relatively lower performance. Notably, the baseline TyG index had the weakest association with MASLD risk (C-index = 0.685, 95% CI 0.677–0.693). To further explore sex differences in predictive performance, we conducted time-dependent ROC analyses stratified by sex. We found that cardiometabolic indices had a higher AUC in predicting the onset of MASLD in females and TyG-BMI had the highest predictive value (Fig. S13).

## Discussion

In this large population cohort study, we investigated the associations between cardiometabolic indices and the incidence of MASLD as well as its progression to liver fibrosis. There was a nonlinear association between cardiometabolic indices and MASLD. High levels of cardiometabolic indices are independently associated with an increased risk of MASLD. Among the indicators, TyG-BMI showed the strongest correlation with MASLD as well as liver fibrosis and the best predictive ability. After demonstrating the positive association between cardiometabolic indices and MASLD, mediation analyses were performed and found that WC and BMI mainly mediated these correlations. The subgroup analyses showed that in each subgroup stratified, the positive associations between cardiometabolic indices and MASLD were consistent, however, there was an interaction between subgroups.

There have been many studies on the relationship between cardiometabolic indices and MASLD, however, most were cross-sectional with small sample sizes and focused on a single cardiometabolic index, the conclusions were inconsistent. No study has been conducted comparing these several metabolism-related indicators. CMI, as a metabolic indicator consisting of blood lipid and obesity parameters, was initially used to distinguish diabetic [13]. Subsequent studies found that CMI was closely associated with hypertension and ischemic stroke [35, 36]. However, studies on the relationship of CMI with fatty liver and liver fibrosis have drawn inconsistent conclusions. A cross-sectional study using NHANES data, illustrated a positive correlation between CMI and both NAFLD and liver fibrosis [37]. Cheng et al. demonstrated that CMI was associated with hepatic steatosis and this association was stronger in the female than in the male, but CMI was not associated with liver fibrosis [38]. This observation align with the results of the current study, potentially due to variations in fat distribution between genders and the effect of sex hormones [39]. AIP is a novel index for assessing blood lipid levels [40]. Duan et al. observed that the risk of MAFLD increased by 12.42-fold for every 1-SD increase in AIP after adjusting for confounders [41]. A study using the Dryad database indicated an association of AIP with new-onset NAFLD in non-obese populations. This correlation was found to be independent of age and sex, but varied across BMI groups [42]. However, in this study, we found that the association between AIP and MASLD was stronger in young, female. As an alternative biomarker of insulin resistance, the TyG index has been reported to be associated with the onset and prognosis of numerous cardiovascular diseases, such as in-stent restenosis, acute coronary syndrome, heart failure, coronary artery calcification and stroke [43–47]. Recent investigations have examined the relationship between the TyG index and its derivatives in relation to MASLD. A cross-sectional study of 230 patients with NAFLD in Iran suggested that the TyG index was positively correlated with liver fibrosis as defined by the NAFLD fibrosis score and fibrosis-4 [48]. Zhang et al. included 393 individuals diagnosed with NAFLD by liver biopsy, observed that ORs for NASH, significant fibrosis, advanced fibrosis and cirrhosis were higher in the fourth quartile of TyG-BMI [49]. Song et al. indicated that the TyG index and modified TyG indices were associated with NAFLD in children and adolescents [50].

Although our study confirms that cardiometabolic indices are associated with the new-onset of MASLD, the biological processes that mediate these associations have not been fully elucidated. The mediation analyses of this study revealed that this may be mediated by obesity. Under conditions of prolonged caloric surplus, surplus nutrients are initially sequestered as lipid deposits within adipose tissue, driving adiposity [51]. Adipose tissue dysfunction in obesity triggers the release of pro-inflammatory cytokines (e.g., TNF-α, IL-6) and reduces adiponectin, an anti-inflammatory and insulin-sensitizing adipokine [52]. This imbalance promotes systemic inflammation that ultimately leads to insulin resistance (IR) [52]. Sustained nutrient loading drives excess free fatty acids (FFAs) to deposit ectopically in non-adipose organs such as the liver, where they overwhelm hepatic β-oxidation capacity, culminating in triglyceride accumulation within hepatocytes—the hallmark of hepatic steatosis [51, 53]. In addition, this ectopic fat accumulation elevated reactive oxygen species (ROS) generation, and systemic low-grade inflammation [51]. Critically, obesity-associated chronic inflammation disrupts insulin signaling pathways, serving as a central mechanism in the pathogenesis of insulin resistance [51]. Obesity is a key contributor to IR and FFA overflow. Bo et al. found that under insulin-resistant conditions, insulin's ability to suppress gluconeogenesis was impaired, leading to increased hepatic glucose output, while its capacity to promote lipogenesis was not significantly impaired. Second, although insulin's direct stimulation of hepatic lipid synthesis was reduced in insulin resistance, the elevated hepatic glucose output provides substrates that drive hepatic lipid synthesis, ultimately leading to increased hepatic lipid accumulation. Third, extrahepatic factors significantly contribute to hepatic lipid deposition in insulin-resistant states. These include elevated adipose tissue lipolysis and reduced skeletal muscle glucose uptake, which result in increased circulating levels of glucose, free fatty acids, and glycerol. These metabolites further promote hepatic gluconeogenesis and lipid synthesis. [11]. Our findings that TyG-BMI demonstrated the strongest association with MASLD and liver fibrosis align with these mechanisms. The superior predictive performance of TyG-BMI likely stems from its dual incorporation of lipid-glucose dysregulation (TyG) and adiposity (BMI), capturing both IR severity and its fat-mediated effects.

### Strengths and limitations

First, this study utilized large cohort population data to compare six indices systematically—CMI, AIP, TyG, TyG-BMI, TyG-WC, and TyG-WHtR—for predicting MASLD incidence and its progression to liver fibrosis. Our results demonstrate that TyG-BMI outperforms other markers in predicting MASLD onset and its progression to liver fibrosis, with superior time-dependent AUC. This multi-index comparison fills a gap in identifying optimal non-invasive tools for early MASLD detection, providing valuable references for early detection, reversal of liver steatosis, and avoidance of more serious health problems. Second, this study revealed that obesity may mediate the association between cardiovascular indices and MASLD, suggesting that managing obesity and lipid levels may help prevent MASLD, highlighting the potential value of cardiometabolic indices as indicators of early intervention. It also provides a focus for the health management of high-risk groups. Third, we established multiple models and performed multiple subgroup and sensitivity analyses, the results were convincing. As a simple composite marker integrating triglycerides, glucose, and obesity, TyG-BMI is feasible for rapid risk stratification in primary care. There were also several limitations in this study. First, lifestyle factors like diet and physical exercise are crucial in the progression of MASLD, yet their impact was not considered in this study. Second, only cardiometabolic indices at baseline were considered and the associations of cardiometabolic indices over time with MASLD and fibrosis were not evaluated. The well-established measure of insulin resistance, HOMA-IR, was also not included in the analysis. Third, in this study, fatty liver was diagnosed through ultrasound examination, and liver fibrosis was assessed using a noninvasive score. Although not the gold standard for diagnosis, it is the most commonly used method in large-scale epidemiological surveys. Furthermore, due to the relatively short follow-up period and the sample size insufficient to achieve robust statistical power, we could not determine which cardiometabolic index had the highest predictive value for major adverse liver outcomes including hepatocellular carcinoma, liver cirrhosis and its complications and liver-related deaths. Finally, the population of this study was derived from China, the generalizability of the conclusions needs to be further confirmed in other races or groups.

## Conclusions

Our findings revealed that the risk of developing MASLD increases as the level of cardiometabolic indices increases. Obesity may serve as a mediating factor in the aforementioned association. In addition, TyG-BMI showed the strongest association with the onset of MASLD and its progression to liver fibrosis, proved to be superior to other indicators, and may be the most appropriate clinical non-invasive biomarker for predicting the onset of MASLD and liver fibrosis, providing references for early identification and reversal of liver steatosis and avoidance of more serious health problems.

## Supplementary Information

Additional file1.

## Data Availability

No datasets were generated or analysed during the current study.

## Abbreviations

MASLD: Metabolic dysfunction-associated steatotic liver disease
CMI: Cardiometabolic index
AIP: Atherogenic index of plasma
TyG: Triglyceride glucose
TyG-BMI: Triglyceride glucose-body mass index
TyG-WC: Triglyceride glucose-waist circumference
TyG-WHtR: Triglyceride glucose-waist height ratio
HR: Hazard ratio
CI: Confidence interval
ROC: Receiver operating characteristic
BMI: Body mass index
WC: Waist circumference
SBP: Systolic blood pressure
DBP: Diastolic blood pressure
WBC: White blood cell
RBC: Red blood cell
ALT: Alanine aminotransferase
AST: Aspartate aminotransferase
GGT: Gamma-glutamyl transpeptidase
SUA: Serum uric acid
Scr: Serum creatinine
TC: Total cholesterol
TG: Triglyceride
HDL-C: High-density lipoprotein cholesterol
LDL-C: Low-density lipoprotein cholesterol
FPG: Fasting plasma glucose
RCS: Restricted cubic spline
PSM: Propensity score matching
NFS: NAFLD fibrosis score
MAF-5: Metabolic Dysfunction-Associated Fibrosis 5
