# Supplementary material for: Associations between cardiometabolic indices and the onset of metabolic dysfunction-associated steatotic liver disease as well as its progression to liver fibrosis: a cohort study
Source: Cardiovasc Diabetol. 2025 Apr 3;24:154. doi: 10.1186/s12933-025-02716-6 (PMC11969729; doi:10.1186/s12933-025-02716-6)
Supplement: Supplementary file 1 — Additional file1. [file 12933_2025_2716_MOESM1_ESM.docx]

**Supplementary Materials**

The calculation formula of cardiometabolic indices are as follows:

1. CMI=TG (mmol/L)/HDL-C (mmol/L)×WC (cm)/height (cm)^[1]^;
2. AIP=log10[TG(mmol/L)/HDL-C (mmol/L)]^[2]^;
3. TyG=ln[TG (mg/dL)×FPG (mg/dL)/2 ]^[3]^;
4. TyG-BMI= TyG×BMI;
5. TyG-WC=TyG×WC;
6. TyG-WHtR=TyG×WHtR

[1] Wakabayashi I, Daimon T. The "cardiometabolic index" as a new marker determined by adiposity and blood lipids for discrimination of diabetes mellitus [J]. Clin Chim Acta, 2015, 438: 274-278.

[2] Huang Q, Liu Z, Wei M, et al. The atherogenic index of plasma and carotid atherosclerosis in a community population: a population-based cohort study in China [J]. Cardiovasc Diabetol, 2023, 22(1): 125.

[3] Zhou Q, Yang J, Tang H, et al. High triglyceride-glucose (TyG) index is associated with poor prognosis of heart failure with preserved ejection fraction [J]. Cardiovasc Diabetol, 2023, 22(1): 263.

**Figure S1.** Flow chart of study subjects

**Figure S2.** Summary of missing variables

**Figure S3.** The final constructed directed acyclic graph

**Figure S4.** Cumulative incidence curves for incident MASLD stratified by cardiometabolic indices

**Figure S5.** Restricted cubic spline analysis of cardiometabolic biomarkers with liver fibrosis

**Figure S6.** Distribution of covariates before and after propensity score matching between high CMI and low CMI group

**Figure S7.** Distribution of covariates before and after propensity score matching between high AIP and low AIP group

**Figure S8.** Distribution of covariates before and after propensity score matching between high TyG and low TyG group

**Figure S9.** Distribution of covariates before and after propensity score matching between high TyG-BMI and low TyG-BMI group

**Figure S10.** Distribution of covariates before and after propensity score matching between high TyG-WC and low TyG-WC group

**Figure S11.** Distribution of covariates before and after propensity score matching between high TyG-WHtR and low TyG-WHtR group

**Figure S12.** Time-dependent ROC curves of cardiometabolic indices for predicting the onset of MASLD

**Figure S13.** Time-dependent ROC curves of cardiometabolic indices for predicting the onset of MASLD in males (A) and females (B)

**Table S1.** Baseline characteristics of the study population according to cardiometabolic index (CMI) quartiles

**Table S2.** Baseline characteristics of the study population according to atherogenic index of plasma (AIP) quartiles

**Table S3.** Baseline characteristics of the study population according to triglyceride-glucose index (TyG) quartiles

**Table S4.** Baseline characteristics of the study population according to triglyceride-glucose × body mass index (TyG-BMI) quartiles

**Table S5.** Baseline characteristics of the study population according to triglyceride-glucose × waist circumference (TyG-WC) quartiles

**Table S6.** Baseline characteristics of the study population according to triglyceride-glucose × waist circumference/height (TyG-WHtR) quartiles

**Table S7.** Comparison of the incidence rate and incidence density of MASLD in different groups

**Table S8.** Association between cardiometabolic indices and MASLD components

**Table S9.** Baseline characteristics of the study population with and without liver fibrosis

**Table S10.** Association between cardiometabolic indices and non-invasive fibrosis score

**Table S11.** Association between cardiometabolic index (CMI) and MASLD stratified by age, sex, BMI, hypertension, diabetes and dyslipidemia status

**Table S12.** Association between atherogenic index of plasma (AIP) and MASLD stratified by age, sex, BMI, hypertension, diabetes and dyslipidemia status

**Table S13.** Association between triglyceride-glucose index (TyG) and MASLD stratified by age, sex, BMI, hypertension, diabetes and dyslipidemia status

**Table S14.** Association between triglyceride-glucose × body mass index (TyG-BMI) and MASLD stratified by age, sex, BMI, hypertension, diabetes and dyslipidemia status

**Table S15.** Association between triglyceride-glucose × waist circumference (TyG-WC) and MASLD stratified by age, sex, BMI, hypertension, diabetes and dyslipidemia status

**Table S16.** Association between triglyceride-glucose × waist circumference/height (TyG-WHtR) and MASLD stratified by age, sex, BMI, hypertension, diabetes and dyslipidemia status

**Table S17.** Associations between cardiometabolic indices and the incidence of MASLD after excluding individuals with MASLD occurring within 1 year of follow-up

**Table S18.** Associations between cardiometabolic indices and the incidence of MASLD after excluding individuals who used antihypertensive, hypoglycemic or lipid-lowing drugs

**Table S19.** Associations between cardiometabolic indices and the incidence of MASLD after grouping by optimal cutoff point

**Table S20.** Associations between cardiometabolic indices and the incidence of MASLD after propensity score matching

**Table S21.** Analysis of the mediation between cardiometabolic indices and MASLD


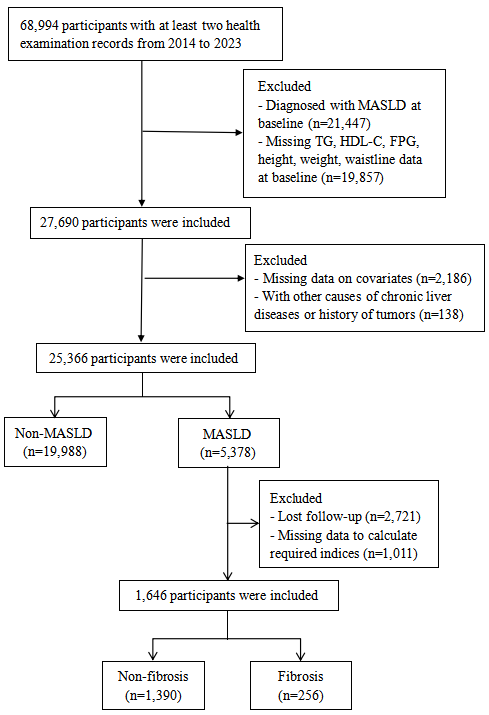


**Figure S1.** Flow chart of study subjects


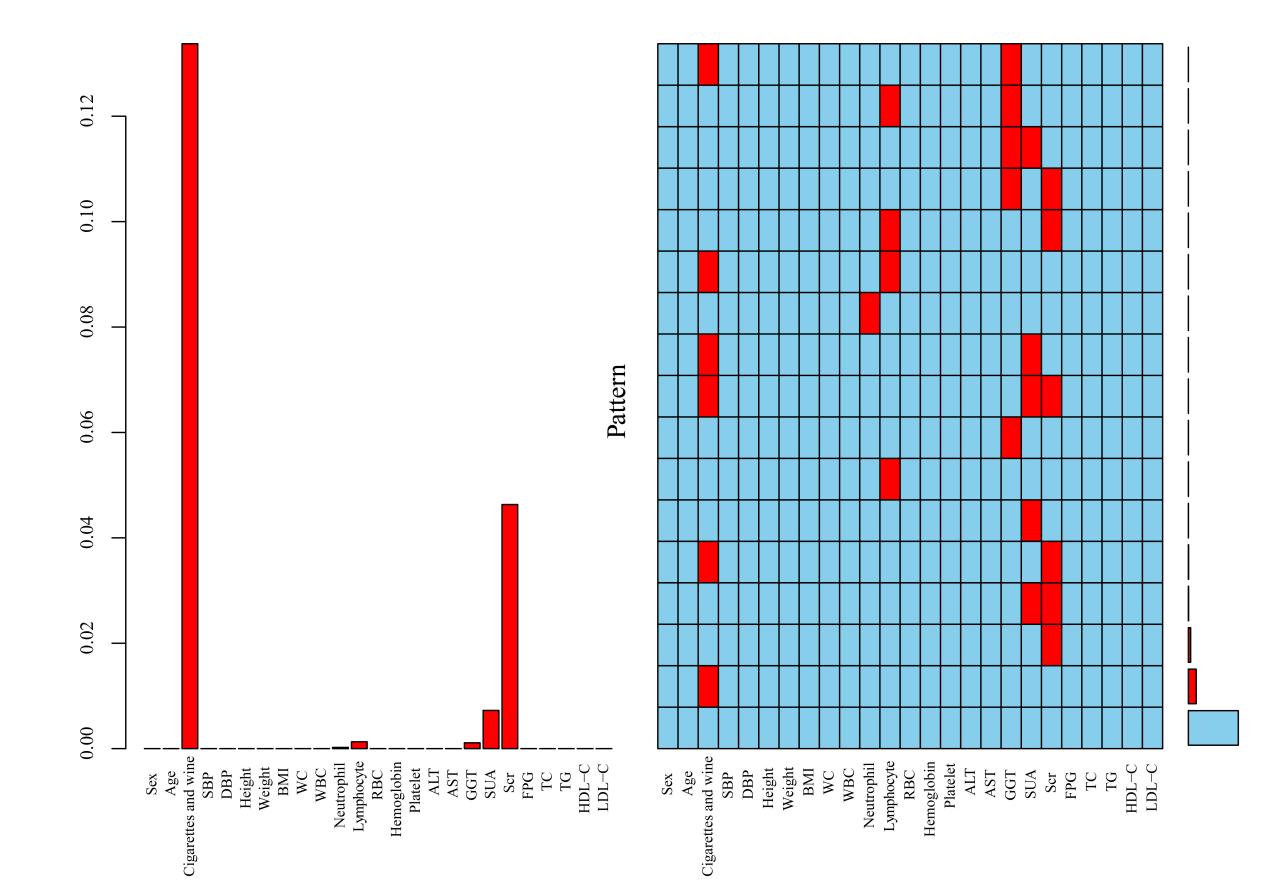


**Figure S2.** Summary of missing variables

The missing proportion of smoking and drinking status, neutrophils, lymphocyte, GGT, SUA and Scr was 13.38%, 0.02%, 0.13%, 0.11%, 0.73% and 4.63%, respectively.

Abbreviations: SBP systolic blood pressure, DBP diastolic blood pressure, BMI body mass index, WC waist circumference, WBC white blood cell, RBC red blood cell, ALT alanine aminotransferase, AST aspartate aminotransferase, GGT γ-glutamyl transpeptidase, SUA serum uric acid, Scr serum creatinine, FPG fasting plasma glucose, TC total cholesterol, TG triglyceride, HDL-C high-density lipoprotein cholesterol, LDL-C low-density lipoprotein cholesterol


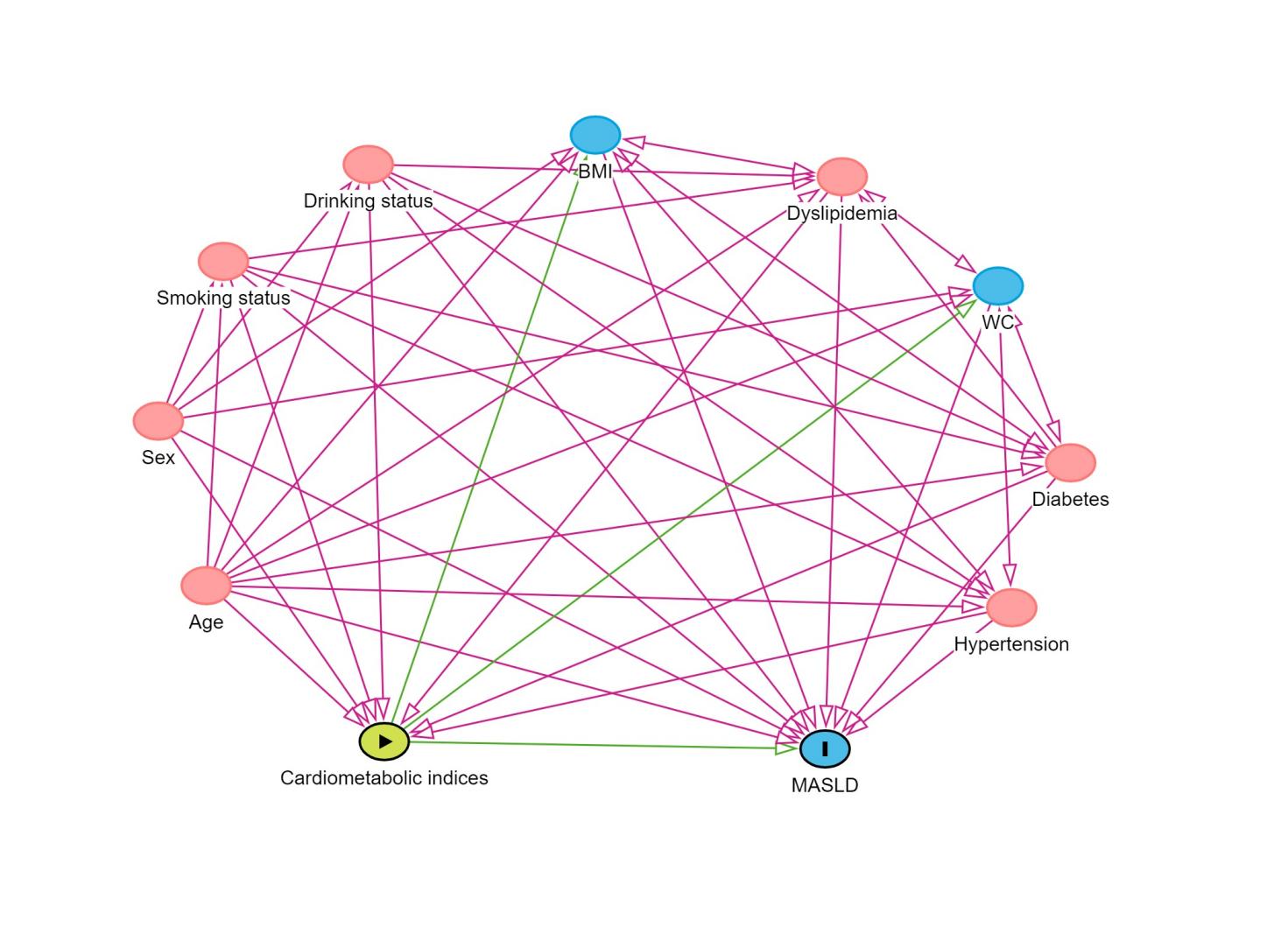


**Figure S3.** The final constructed directed acyclic graph

Abbreviations: BMI body mass index, WC waist circumference, MASLD metabolic dysfunction-associated steatotic liver disease


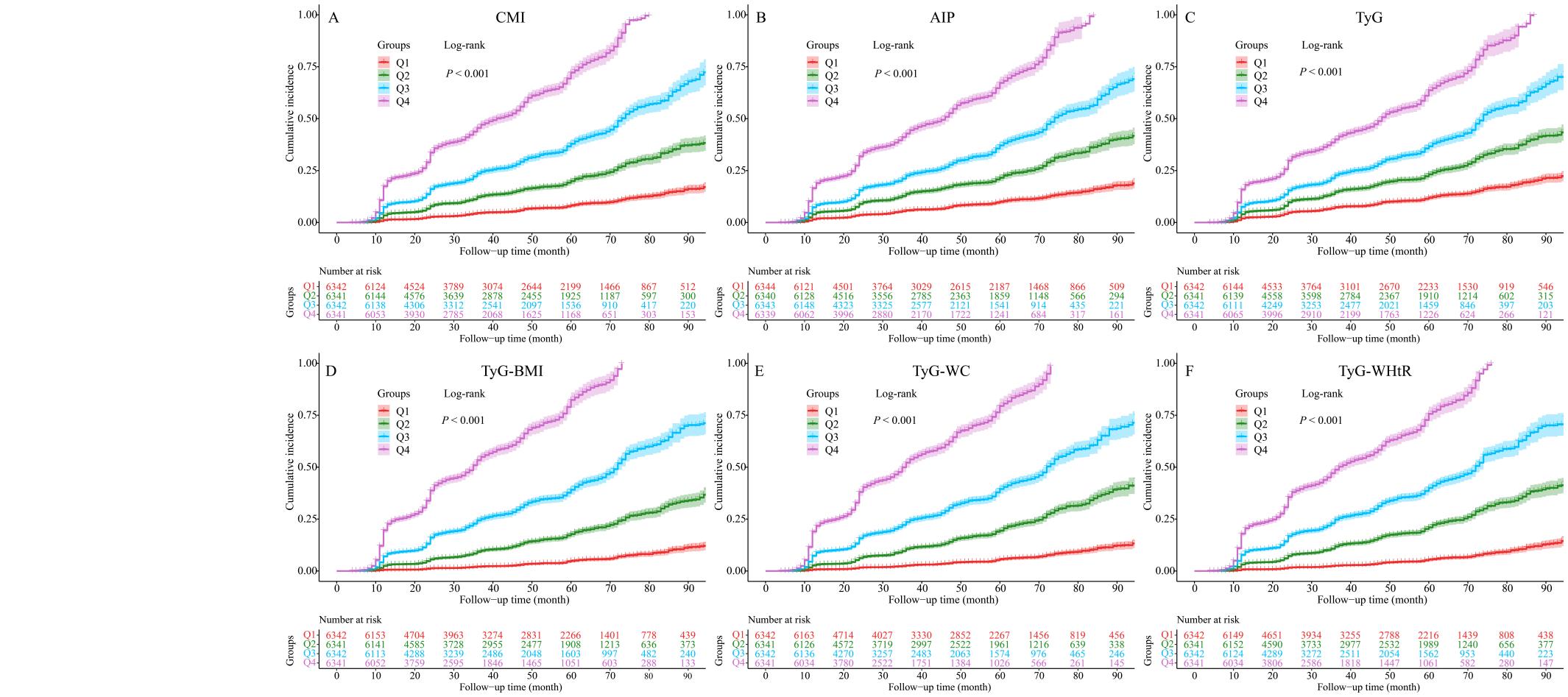


**Figure S4.** Cumulative incidence curves for incident MASLD stratified by cardiometabolic indices

Abbreviation: CMI cardiometabolic index, AIP atherogenic index of plasma, TyG triglyceride-glucose index, TyG-BMI triglyceride-glucose × body mass index, TyG-WC triglyceride-glucose × waist circumference, TyG-WHtR triglyceride-glucose × waist circumference/height, MASLD metabolic dysfunction-associated steatotic liver disease


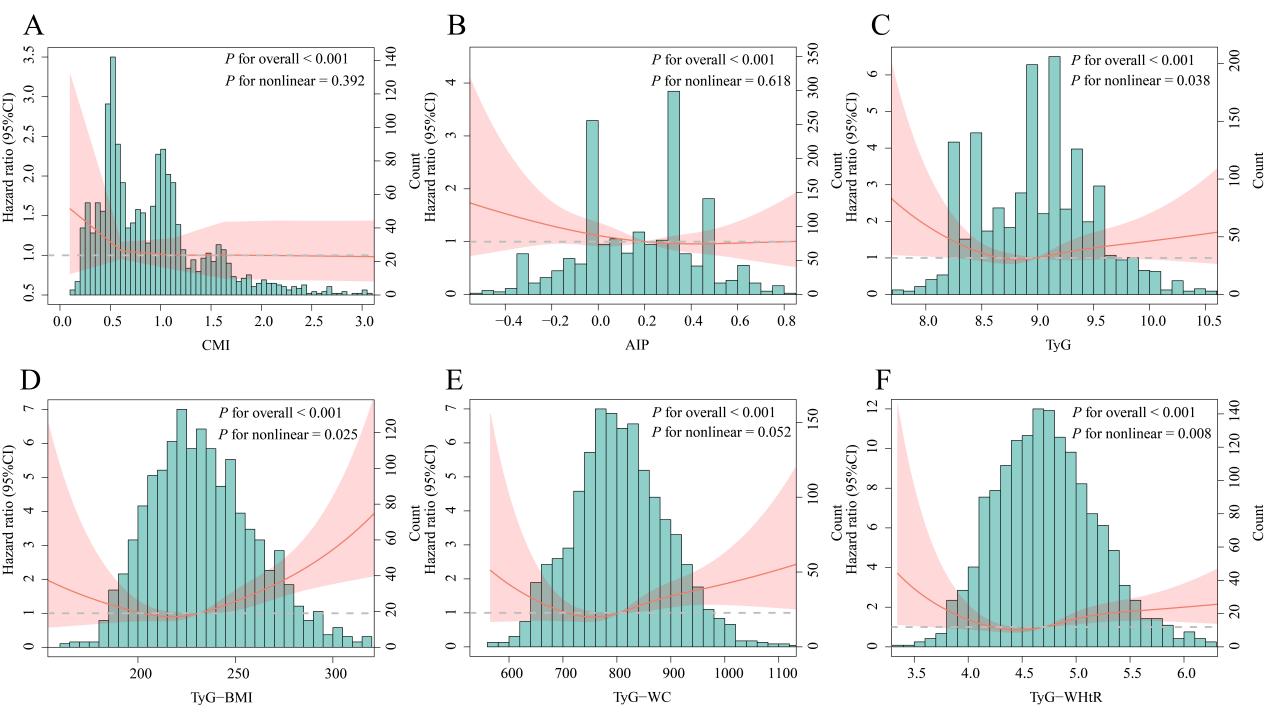


**Figure S5.** Restricted cubic spline analysis of cardiometabolic biomarkers with liver fibrosis

CMI cardiometabolic index, AIP atherogenic index of plasma, TyG triglyceride-glucose index, TyG-BMI triglyceride-glucose × body mass index, TyG-WC triglyceride-glucose × waist circumference, TyG-WHtR triglyceride-glucose × waist circumferece/height


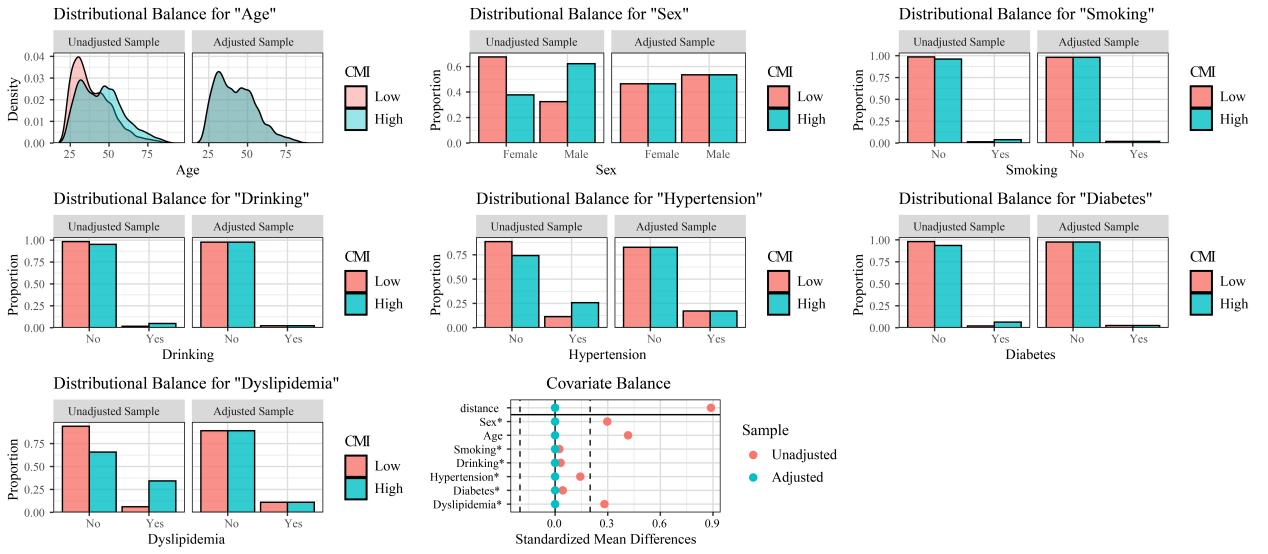


**Figure S6.** Distribution of covariates before and after propensity score matching between high CMI and low CMI group

CMI cardiometabolic index


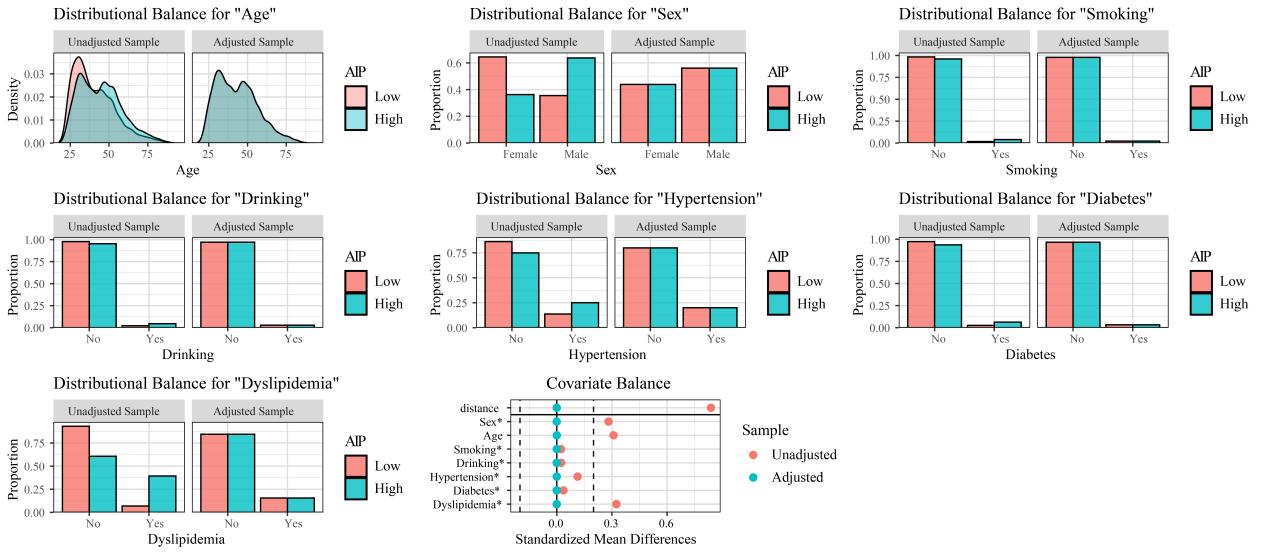


**Figure S7.** Distribution of covariates before and after propensity score matching between high AIP and low AIP group

AIP atherogenic index of plasma


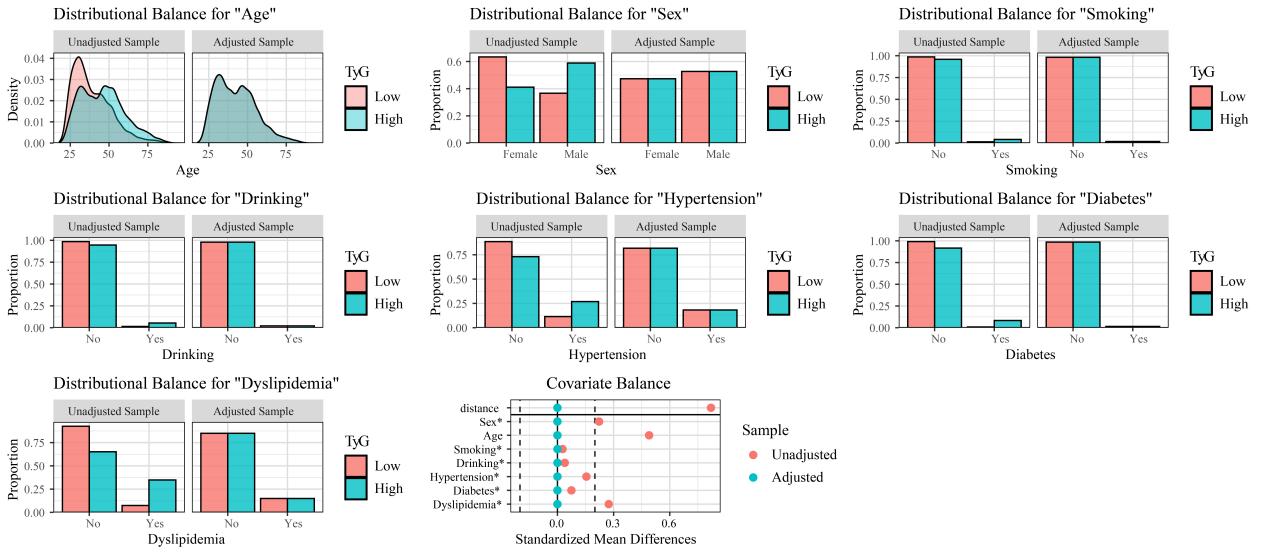


**Figure S8.** Distribution of covariates before and after propensity score matching between high TyG and low TyG group

TyG triglyceride-glucose index


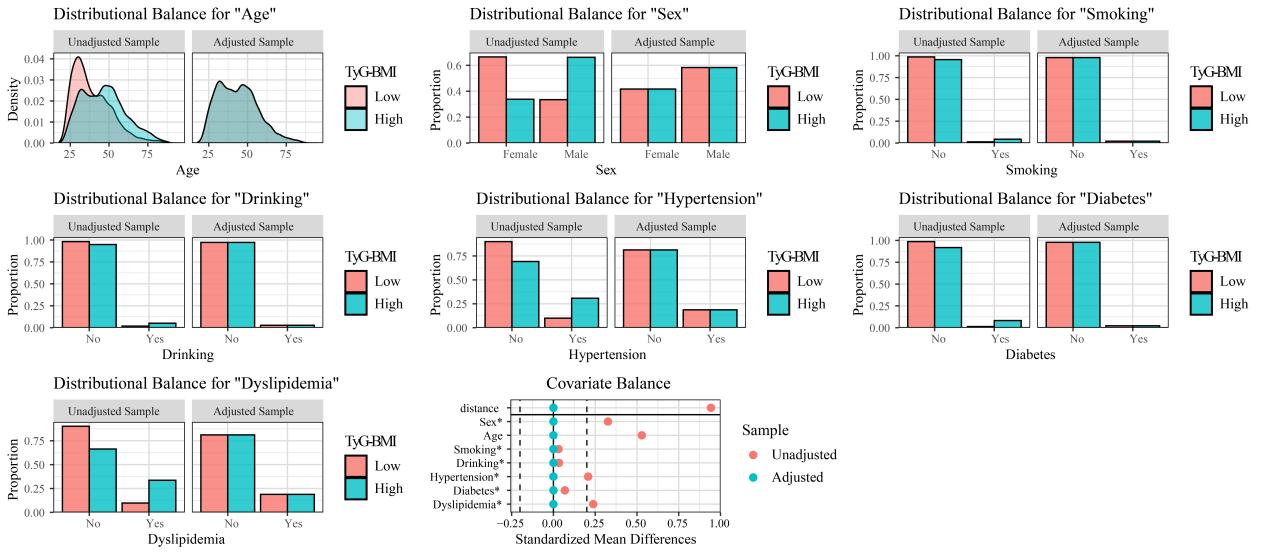


**Figure S9.** Distribution of covariates before and after propensity score matching between high TyG-BMI and low TyG-BMI group

TyG-BMI triglyceride-glucose × body mass index


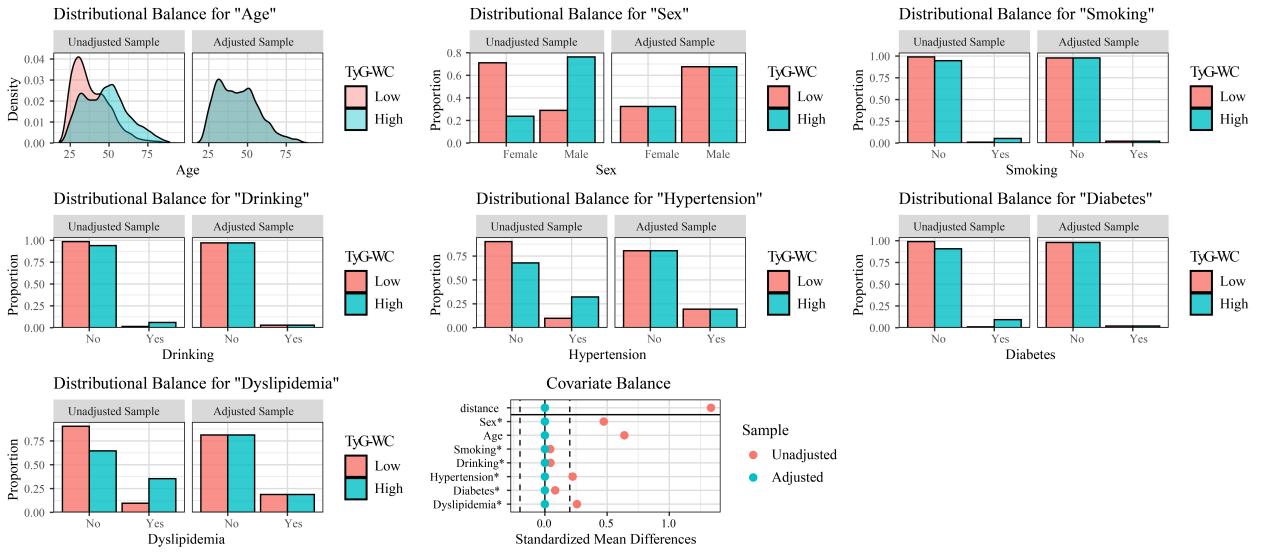


**Figure S10.** Distribution of covariates before and after propensity score matching between high TyG-WC and low TyG-WC group

TyG-WC triglyceride-glucose × waist circumference


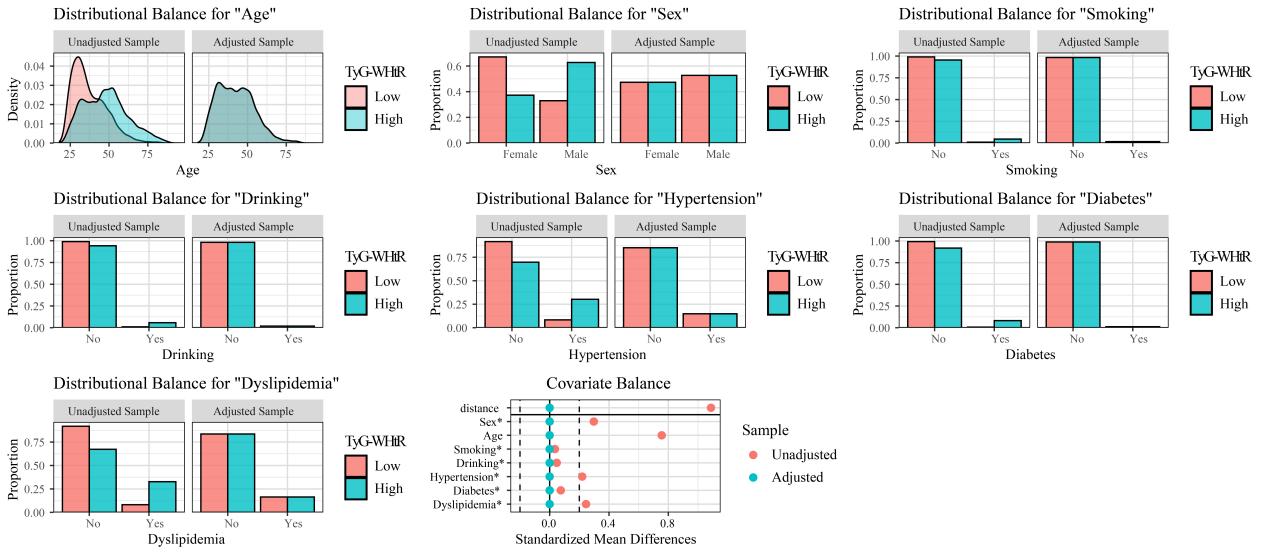


**Figure S11.** Distribution of covariates before and after propensity score matching between high TyG-WHtR and low TyG-WHtR group

TyG-WHtR triglyceride-glucose × waist circumference/height





**Figure S12.** Time-dependent ROC curves of cardiometabolic indices for predicting the onset of MASLD

ROC receiver operating characteristic curve, CMI cardiometabolic index, AIP atherogenic index of plasma, TyG triglyceride-glucose index, TyG-BMI triglyceride-glucose × body mass index, TyG-WC triglyceride-glucose × waist circumference, TyG-WHtR triglyceride-glucose × waist circumference/height

**
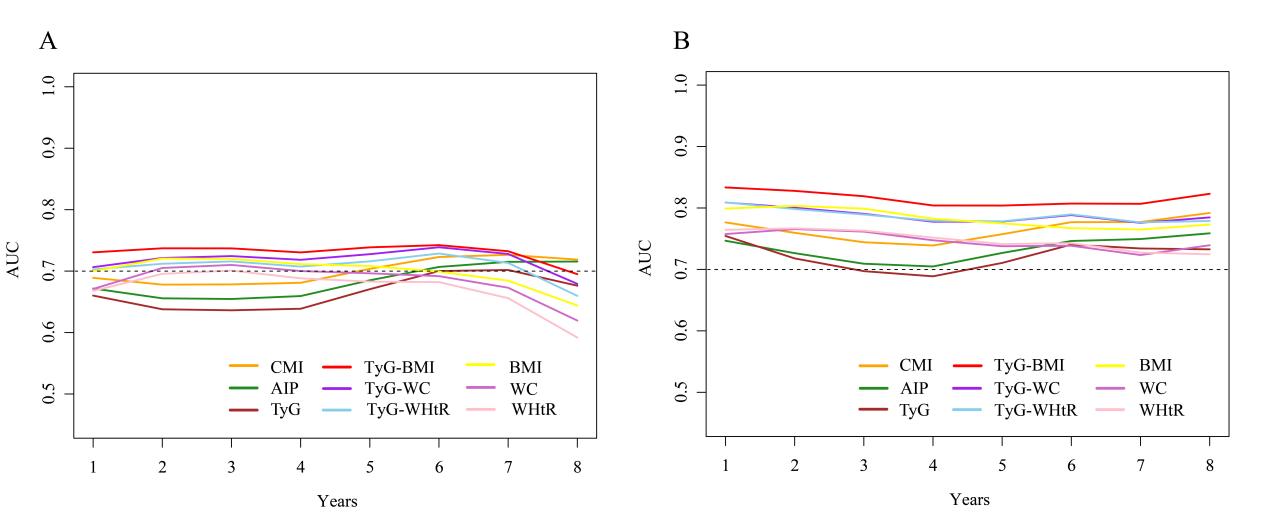
**

**Figure S13.** Time-dependent ROC curves of cardiometabolic indices for predicting the onset of MASLD in males (A) and females (B)

ROC receiver operating characteristic curve, CMI cardiometabolic index, AIP atherogenic index of plasma, TyG triglyceride-glucose index, TyG-BMI triglyceride-glucose × body mass index, TyG-WC triglyceride-glucose × waist circumference, TyG-WHtR triglyceride-glucose × waist circumference/height

**Table S1.** Baseline characteristics of the study population according to cardiometabolic index (CMI) quartiles

| Variables | Q1 (0.08~0.26) (n=6342) | Q2 (0.26~0.40)  (n=6341) | Q3 (0.40~0.62)  (n=6342) | Q4 (0.62~6.12) (n=6341) | *P* value |
| --- | --- | --- | --- | --- | --- |
| Sex, male, n (%) | 1465 (23.10) | 2489 (39.25) | 3284 (51.78) | 4416 (69.64) | <0.001 |
| Age, years | 34.00 (28.00, 44.00) | 38.00 (30.00, 49.00) | 42.00 (32.00, 53.00) | 46.00 (35.00, 55.00) | <0.001 |
| Smoking status, n (%) | 45 (0.71) | 127 (2.00) | 166 (2.62) | 306 (4.83) | <0.001 |
| Drinking status, n (%) | 58 (0.91) | 146 (2.30) | 238 (3.75) | 344 (5.43) | <0.001 |
| BMI, kg/m^2^ | 21.25±2.45 | 22.62±2.68 | 23.70±2.70 | 24.98±2.65 | <0.001 |
| WC, cm | 74.06±7.75 | 79.07±8.42 | 82.73±8.47 | 87.32±8.07 | <0.001 |
| SBP, mmHg | 118.68±14.40 | 122.60±15.67 | 125.88±16.57 | 130.34±17.26 | <0.001 |
| DBP, mmHg | 71.48±9.35 | 73.61±10.12 | 75.52±10.35 | 78.57±11.07 | <0.001 |
| WBC, 10^9^/L | 5.50±1.31 | 5.77±1.36 | 6.04±1.43 | 6.38±1.54 | <0.001 |
| Neutrophils, 10^9^/L | 3.20±1.05 | 3.38±1.08 | 3.55±1.12 | 3.77±1.19 | <0.001 |
| Lymphocytes, 10^9^/L | 1.88±0.49 | 1.94±0.51 | 2.01±0.54 | 2.10±0.57 | <0.001 |
| RBC, 10^12^/L | 4.56±0.38 | 4.70±0.42 | 4.80±0.43 | 4.95±0.43 | <0.001 |
| Hemoglobin, g/L | 135.14±14.31 | 139.96±15.46 | 143.71±15.42 | 149.40±14.65 | <0.001 |
| Platelet, 10^9^/L | 237.16±51.14 | 239.45±52.88 | 240.06±54.42 | 236.43±53.66 | <0.001 |
| FPG, mmol/L | 5.22 (4.98, 5.50) | 5.33 (5.07, 5.63) | 5.41 (5.14, 5.75) | 5.54 (5.24, 5.92) | <0.001 |
| TC, mmol/L | 4.65±0.82 | 4.73±0.86 | 4.85±0.89 | 5.00±0.92 | <0.001 |
| TG, mmol/L | 0.70 (0.59, 0.82) | 0.99 (0.87, 1.15) | 1.32 (1.15, 1.51) | 1.98 (1.66, 2.46) | <0.001 |
| HDL-C, mmol/L | 1.68±0.29 | 1.47±0.25 | 1.34±0.24 | 1.16±0.21 | <0.001 |
| LDL-C, mmol/L | 2.30±0.62 | 2.51±0.66 | 2.68±0.68 | 2.77±0.69 | <0.001 |
| ALT, U/L | 14.00 (11.00, 18.04) | 15.45 (11.94, 21.00) | 17.10 (13.00, 23.33) | 20.43 (15.39, 28.00) | <0.001 |
| AST,U/L | 18.00 (15.57, 20.99) | 18.20 (15.83, 21.79) | 19.00 (16.23, 22.31) | 20.00 (17.04, 23.81) | <0.001 |
| GGT, U/L | 11.42 (9.00, 15.14) | 13.22 (10.07, 18.93) | 15.78 (11.79, 23.00) | 20.32 (14.55, 31.73) | <0.001 |
| SUA, μmol/L | 287.74±70.48 | 312.10±77.79 | 334.76±81.68 | 369.75±84.16 | <0.001 |
| Scr, μmol/L | 57.44 (51.39, 66.32) | 61.60 (53.65, 73.78) | 66.19 (55.95, 77.66) | 71.44 (60.72, 81.00) | <0.001 |
| AIP | -0.37±0.10 | -0.16±0.06 | 0.00±0.06 | 0.26±0.15 | <0.001 |
| TyG | 7.98±0.26 | 8.36±0.23 | 8.67±0.24 | 9.14±0.35 | <0.001 |
| TyG-BMI | 169.56±19.94 | 189.11±21.82 | 205.41±22.99 | 228.41±25.46 | <0.001 |
| TyG-WC | 590.88±63.34 | 660.91±68.22 | 717.09±72.23 | 798.53±79.75 | <0.001 |
| TyG-WHtR | 3.56±0.36 | 3.95±0.38 | 4.26±0.40 | 4.70±0.45 | <0.001 |
| Hypertension, n (%) | 503 (7.93) | 868 (13.69) | 1280 (20.18) | 1891 (29.82) | <0.001 |
| Diabetes, n (%) | 81 (1.28) | 154 (2.43) | 280 (4.42) | 494 (7.79) | <0.001 |
| Dyslipidemia , n (%) | 290 (4.57) | 444 (7.00) | 792 (12.49) | 3263 (51.46) | <0.001 |

The data are presented as the means ± SDs, n (%), or medians (quartile 1, quartile 3).

Abbreviations: MASLD metabolic dysfunction-associated steatotic liver disease, BMI body mass index, WC waist circumference, SBP systolic blood pressure, DBP diastolic blood pressure, WBC white blood cell, RBC red blood cell, FPG fasting plasma glucose, TC total cholesterol, TG triglyceride, HDL-C high-density lipoprotein cholesterol, LDL-C low-density lipoprotein cholesterol, ALT alanine aminotransferase, AST aspartate aminotransferase, GGT gamma-glutamyl transpeptidase, SUA serum uric acid, Scr serum creatinine, AIP atherogenic index of plasma, TyG triglyceride-glucose index, TyG-BMI triglyceride-glucose × body mass index, TyG-WC triglyceride-glucose × waist circumference, TyG-WHtR triglyceride-glucose × waist circumference/height

**Table S2.** Baseline characteristics of the study population according to atherogenic index of plasma (AIP) quartiles

| Variables | Q1 (-0.74~-0.25) (n=6344) | Q2 (-0.25~-0.08)  (n=6340) | Q3 (-0.08~0.10)  (n=6343) | Q4 (0.10~1.03) (n=6339) | *P* value |
| --- | --- | --- | --- | --- | --- |
| Sex, male, n (%) | 1569 (24.73) | 2515 (39.67) | 3244 (51.14) | 4326 (68.24) | <0.001 |
| Age, years | 35.00 (29.00, 46.00) | 39.00 (30.00, 50.00) | 41.00 (32.00, 52.00) | 45.00 (34.00, 54.00) | <0.001 |
| Smoking status, n (%) | 64 (1.01) | 122 (1.92) | 176 (2.77) | 282 (4.45) | <0.001 |
| Drinking status, n (%) | 82 (1.29) | 148 (2.33) | 255 (4.02) | 301 (4.75) | <0.001 |
| BMI, kg/m^2^ | 21.67±2.64 | 22.73±2.85 | 23.55±2.82 | 24.61±2.71 | <0.001 |
| WC, cm | 75.65±8.40 | 79.41±9.04 | 82.18±8.99 | 85.94±8.53 | <0.001 |
| SBP, mmHg | 119.54±14.95 | 123.04±15.99 | 125.69±16.63 | 129.24±17.10 | <0.001 |
| DBP, mmHg | 71.80±9.55 | 73.87±10.23 | 75.47±10.43 | 78.05±11.01 | <0.001 |
| WBC, 10^9^/L | 5.52±1.32 | 5.78±1.36 | 6.05±1.44 | 6.36±1.54 | <0.001 |
| Neutrophils, 10^9^/L | 3.21±1.05 | 3.38±1.08 | 3.56±1.13 | 3.75±1.19 | <0.001 |
| Lymphocytes, 10^9^/L | 1.88±0.49 | 1.95±0.52 | 2.01±0.53 | 2.09±0.56 | <0.001 |
| RBC, 10^12^/L | 4.57±0.38 | 4.70±0.42 | 4.81±0.43 | 4.95±0.43 | <0.001 |
| Hemoglobin, g/L | 135.42±14.36 | 140.06±15.57 | 143.74±15.30 | 148.99±14.94 | <0.001 |
| Platelet, 10^9^/L | 236.71±51.45 | 239.42±53.29 | 239.88±53.95 | 237.10±53.45 | <0.001 |
| FPG, mmol/L | 5.24 (4.99, 5.52) | 5.34 (5.09, 5.65) | 5.40 (5.13, 5.73) | 5.52 (5.22, 5.89) | <0.001 |
| TC, mmol/L | 4.68±0.82 | 4.74±0.86 | 4.83±0.89 | 4.99±0.91 | <0.001 |
| TG, mmol/L | 0.70 (0.59, 0.81) | 0.99 (0.87, 1.13) | 1.32 (1.17, 1.50) | 2.00 (1.69, 2.47) | <0.001 |
| HDL-C, mmol/L | 1.68±0.29 | 1.47±0.26 | 1.34±0.24 | 1.16±0.21 | <0.001 |
| LDL-C, mmol/L | 2.33±0.62 | 2.52±0.67 | 2.67±0.69 | 2.75±0.69 | <0.001 |
| ALT, U/L | 14.00 (11.00, 18.87) | 15.34 (11.85, 21.00) | 17.00 (13.00, 23.26) | 20.00 (15.02, 27.62) | <0.001 |
| AST,U/L | 18.00 (15.71, 21.00) | 18.22 (15.82, 21.79) | 19.00 (16.17, 22.15) | 20.00 (17.00, 23.58) | <0.001 |
| GGT, U/L | 11.70 (9.13, 15.82) | 13.36 (10.13, 19.00) | 15.50 (11.57, 22.77) | 20.00 (14.03, 31.02) | <0.001 |
| SUA, μmol/L | 289.74±71.39 | 313.13±78.15 | 334.25±82.15 | 367.20±84.97 | <0.001 |
| Scr, μmol/L | 57.61 (51.38, 66.91) | 61.82 (53.93, 73.66) | 65.90 (55.74, 77.44) | 71.19 (60.13, 80.89) | <0.001 |
| CMI | 0.20 (0.16, 0.23) | 0.32 (0.29, 0.36) | 0.49 (0.44, 0.55) | 0.85 (0.71, 1.09) | <0.001 |
| TyG | 7.97±0.26 | 8.37±0.22 | 8.68±0.24 | 9.15±0.34 | <0.001 |
| TyG-BMI | 172.88±22.24 | 190.16±24.52 | 204.28±25.14 | 225.18±26.83 | <0.001 |
| TyG-WC | 603.50±71.80 | 664.36±78.53 | 713.05±81.10 | 786.55±86.80 | <0.001 |
| TyG-WHtR | 3.64±0.41 | 3.97±0.45 | 4.23±0.46 | 4.63±0.49 | <0.001 |
| Hypertension, n (%) | 596 (9.39) | 941 (14.84) | 1274 (20.09) | 1731 (27.31) | <0.001 |
| Diabetes, n (%) | 103 (1.62) | 177 (2.79) | 283 (4.46) | 446 (7.04) | <0.001 |
| Dyslipidemia , n (%) | 303 (4.78) | 467 (7.37) | 758 (11.95) | 3261 (51.44) | <0.001 |

The data are presented as the means ± SDs, n (%), or medians (quartile 1, quartile 3).

Abbreviations: MASLD metabolic dysfunction-associated steatotic liver disease, BMI body mass index, WC waist circumference, SBP systolic blood pressure, DBP diastolic blood pressure, WBC white blood cell, RBC red blood cell, FPG fasting plasma glucose, TC total cholesterol, TG triglyceride, HDL-C high-density lipoprotein cholesterol, LDL-C low-density lipoprotein cholesterol, ALT alanine aminotransferase, AST aspartate aminotransferase, GGT gamma-glutamyl transpeptidase, SUA serum uric acid, Scr serum creatinine, CMI cardiometabolic index, TyG triglyceride-glucose index, TyG-BMI triglyceride-glucose × body mass index, TyG-WC triglyceride-glucose × waist circumference, TyG-WHtR triglyceride-glucose × waist circumference/height

**Table S3.** Baseline characteristics of the study population according to triglyceride-glucose index (TyG) quartiles

| Variables | Q1 (7.17~8.18) (n=6342) | Q2 (8.18~8.51)  (n=6341) | Q3 (8.51~8.87)  (n=6342) | Q4 (8.87~10.67) (n=6341) | *P* value |
| --- | --- | --- | --- | --- | --- |
| Sex, male, n (%) | 1841 (29.03) | 2590 (40.85) | 3209 (50.60) | 4014 (63.30) | <0.001 |
| Age, years | 34.00 (28.00, 43.00) | 38.00 (30.00, 49.00) | 42.00 (32.00, 52.00) | 47.00 (36.00, 56.00) | <0.001 |
| Smoking status, n (%) | 49 (0.77) | 112 (1.77) | 183 (2.89) | 300 (4.73) | <0.001 |
| Drinking status, n (%) | 44 (0.69) | 120 (1.89) | 245 (3.86) | 377 (5.95) | <0.001 |
| BMI, kg/m^2^ | 21.83±2.68 | 22.80±2.85 | 23.52±2.91 | 24.41±2.77 | <0.001 |
| WC, cm | 76.17±8.51 | 79.61±9.05 | 82.00±9.24 | 85.40±8.81 | <0.001 |
| SBP, mmHg | 118.29±13.86 | 122.59±15.41 | 125.82±16.44 | 130.80±17.76 | <0.001 |
| DBP, mmHg | 71.20±9.30 | 73.77±9.97 | 75.69±10.57 | 78.53±10.97 | <0.001 |
| WBC, 10^9^/L | 5.60±1.34 | 5.82±1.36 | 6.00±1.46 | 6.29±1.54 | <0.001 |
| Neutrophils, 10^9^/L | 3.25±1.06 | 3.40±1.07 | 3.53±1.14 | 3.72±1.19 | <0.001 |
| Lymphocytes, 10^9^/L | 1.90±0.50 | 1.96±0.51 | 2.00±0.54 | 2.07±0.57 | <0.001 |
| RBC, 10^12^/L | 4.60±0.40 | 4.72±0.42 | 4.80±0.44 | 4.90±0.44 | <0.001 |
| Hemoglobin, g/L | 136.24±15.18 | 140.39±15.55 | 143.71±15.32 | 147.86±14.99 | <0.001 |
| Platelet, 10^9^/L | 237.43±52.01 | 239.02±52.97 | 239.02±53.49 | 237.63±53.74 | 0.167 |
| FPG, mmol/L | 5.17 (4.92, 5.41) | 5.32 (5.08, 5.60) | 5.42 (5.16, 5.74) | 5.64 (5.32, 6.10) | <0.001 |
| TC, mmol/L | 4.48±0.77 | 4.71±0.83 | 4.92±0.86 | 5.13±0.93 | <0.001 |
| TG, mmol/L | 0.69 (0.59, 0.78) | 0.99 (0.91, 1.08) | 1.35 (1.24, 1.48) | 2.03 (1.76, 2.47) | <0.001 |
| HDL-C, mmol/L | 1.54±0.31 | 1.45±0.30 | 1.38±0.30 | 1.27±0.29 | <0.001 |
| LDL-C, mmol/L | 2.29±0.60 | 2.52±0.65 | 2.69±0.68 | 2.77±0.71 | <0.001 |
| ALT, U/L | 14.00 (11.00, 18.95) | 15.67 (12.00, 21.11) | 17.04 (13.00, 23.29) | 20.00 (15.00, 27.11) | <0.001 |
| AST,U/L | 18.00 (15.47, 21.00) | 18.22 (15.87, 21.70) | 19.00 (16.31, 22.41) | 20.00 (17.10, 23.86) | <0.001 |
| GGT, U/L | 11.66 (9.09, 15.80) | 13.53 (10.25, 19.08) | 15.72 (11.67, 23.00) | 20.00 (13.99, 31.68) | <0.001 |
| SUA, μmol/L | 296.11±74.82 | 315.62±79.71 | 333.60±81.53 | 359.05±87.60 | <0.001 |
| Scr, μmol/L | 58.00 (51.42, 68.86) | 62.00 (53.87, 74.53) | 66.00 (56.00, 77.37) | 69.48 (58.93, 79.52) | <0.001 |
| CMI | 0.20 (0.16, 0.25) | 0.33 (0.27, 0.40) | 0.49 (0.40, 0.59) | 0.83 (0.65, 1.09) | <0.001 |
| AIP | -0.35±0.13 | -0.16±0.11 | 0.00±0.11 | 0.23±0.17 | <0.001 |
| TyG-BMI | 172.96±22.05 | 190.43±24.08 | 204.23±25.60 | 224.87±27.40 | <0.001 |
| TyG-WC | 603.61±70.71 | 664.87±76.69 | 712.07±81.49 | 786.86±89.12 | <0.001 |
| TyG-WHtR | 3.62±0.39 | 3.96±0.42 | 4.23±0.44 | 4.65±0.50 | <0.001 |
| Hypertension, n (%) | 446 (7.03) | 876 (13.81) | 1273 (20.07) | 1947 (30.70) | <0.001 |
| Diabetes, n (%) | 28 (0.44) | 57 (0.90) | 171 (2.70) | 753 (11.88) | <0.001 |
| Dyslipidemia , n (%) | 282 (4.45) | 561 (8.85) | 926 (14.60) | 3020 (47.63) | <0.001 |

The data are presented as the means ± SDs, n (%), or medians (quartile 1, quartile 3).

Abbreviations: MASLD metabolic dysfunction-associated steatotic liver disease, BMI body mass index, WC waist circumference, SBP systolic blood pressure, DBP diastolic blood pressure, WBC white blood cell, RBC red blood cell, FPG fasting plasma glucose, TC total cholesterol, TG triglyceride, HDL-C high-density lipoprotein cholesterol, LDL-C low-density lipoprotein cholesterol, ALT alanine aminotransferase, AST aspartate aminotransferase, GGT gamma-glutamyl transpeptidase, SUA serum uric acid, Scr serum creatinine, CMI cardiometabolic index, AIP atherogenic index of plasma, TyG-BMI triglyceride-glucose × body mass index, TyG-WC triglyceride-glucose × waist circumference, TyG-WHtR triglyceride-glucose × waist circumference/height

**Table S4.** Baseline characteristics of the study population according to triglyceride-glucose × body mass index (TyG-BMI) quartiles

| Variables | Q1 (117.94~174.97) (n=6342) | Q2 (174.97~196.23)  (n=6341) | Q3 (196.23~218.85)  (n=6342) | Q4 (218.85~334.02) (n=6341) | *P* value |
| --- | --- | --- | --- | --- | --- |
| Sex, male, n (%) | 1348 (21.26) | 2365 (37.30) | 3566 (56.23) | 4375 (69.00) | <0.001 |
| Age, years | 32.00 (28.00, 41.00) | 39.00 (31.00, 49.00) | 43.00 (33.00, 53.00) | 47.00 (36.00, 56.00) | <0.001 |
| Smoking status, n (%) | 43 (0.68) | 100 (1.58) | 182 (2.87) | 319 (5.03) | <0.001 |
| Drinking status, n (%) | 47 (0.74) | 150 (2.37) | 216 (3.41) | 373 (5.88) | <0.001 |
| BMI, kg/m^2^ | 19.70±1.36 | 22.12±1.11 | 24.02±1.23 | 26.72±1.99 | <0.001 |
| WC, cm | 71.40±6.23 | 77.94±6.20 | 83.42±6.28 | 90.41±7.08 | <0.001 |
| SBP, mmHg | 116.67±13.16 | 121.42±14.74 | 126.59±16.16 | 132.83±17.40 | <0.001 |
| DBP, mmHg | 70.82±8.82 | 72.82±9.58 | 75.72±10.41 | 79.83±11.09 | <0.001 |
| WBC, 10^9^/L | 5.59±1.37 | 5.78±1.38 | 5.99±1.41 | 6.35±1.53 | <0.001 |
| Neutrophils, 10^9^/L | 3.27±1.11 | 3.37±1.07 | 3.51±1.10 | 3.74±1.18 | <0.001 |
| Lymphocytes, 10^9^/L | 1.89±0.49 | 1.95±0.52 | 2.00±0.53 | 2.09±0.57 | <0.001 |
| RBC, 10^12^/L | 4.57±0.38 | 4.69±0.42 | 4.82±0.43 | 4.94±0.43 | <0.001 |
| Hemoglobin, g/L | 135.08±14.41 | 139.27±15.38 | 144.88±15.22 | 148.97±14.73 | <0.001 |
| Platelet, 10^9^/L | 239.20±51.36 | 240.43±52.79 | 237.56±53.89 | 235.92±54.06 | <0.001 |
| FPG, mmol/L | 5.17 (4.94, 5.41) | 5.32 (5.07, 5.60) | 5.45 (5.18, 5.77) | 5.62 (5.30, 6.04) | <0.001 |
| TC, mmol/L | 4.55±0.81 | 4.75±0.86 | 4.89±0.88 | 5.04±0.90 | <0.001 |
| TG, mmol/L | 0.81 (0.65, 1.03) | 1.03 (0.82, 1.33) | 1.27 (0.99, 1.64) | 1.69 (1.29, 2.25) | <0.001 |
| HDL-C, mmol/L | 1.59±0.31 | 1.46±0.30 | 1.35±0.28 | 1.25±0.27 | <0.001 |
| LDL-C, mmol/L | 2.28±0.62 | 2.52±0.65 | 2.68±0.67 | 2.78±0.69 | <0.001 |
| ALT, U/L | 13.00 (10.35, 17.00) | 15.46 (12.00, 20.64) | 17.94 (13.81, 24.30) | 21.00 (16.00, 28.26) | <0.001 |
| AST,U/L | 17.43 (15.18, 20.01) | 18.31 (15.88, 21.57) | 19.21 (16.61, 22.89) | 20.20 (17.28, 24.00) | <0.001 |
| GGT, U/L | 11.10 (9.00, 14.75) | 13.01 (10.00, 18.20) | 16.28 (12.00, 23.50) | 21.00 (14.87, 32.68) | <0.001 |
| SUA, μmol/L | 284.15±68.00 | 308.69±76.41 | 340.20±80.76 | 371.48±84.38 | <0.001 |
| Scr, μmol/L | 57.00 (51.22, 65.07) | 61.24 (53.35, 73.06) | 67.34 (56.81, 78.18) | 71.75 (60.85, 81.31) | <0.001 |
| CMI | 0.22 (0.17, 0.30) | 0.34 (0.26, 0.45) | 0.47 (0.35, 0.64) | 0.74 (0.52, 1.02) | <0.001 |
| AIP | -0.28±0.18 | -0.13±0.19 | -0.02±0.20 | 0.15±0.23 | <0.001 |
| TyG | 8.13±0.36 | 8.41±0.38 | 8.64±0.40 | 8.98±0.46 | <0.001 |
| TyG-WC | 580.61±54.67 | 654.99±54.89 | 720.05±56.71 | 811.76±72.70 | <0.001 |
| TyG-WHtR | 3.49±0.31 | 3.92±0.29 | 4.27±0.30 | 4.79±0.41 | <0.001 |
| Hypertension, n (%) | 322 (5.08) | 697 (10.99) | 1288 (20.31) | 2235 (35.25) | <0.001 |
| Diabetes, n (%) | 35 (0.55) | 99 (1.56) | 218 (3.44) | 657 (10.36) | <0.001 |
| Dyslipidemia , n (%) | 322 (5.08) | 698 (11.01) | 1264 (19.93) | 2505 (39.50) | <0.001 |

The data are presented as the means ± SDs, n (%), or medians (quartile 1, quartile 3).

Abbreviations: MASLD metabolic dysfunction-associated steatotic liver disease, BMI body mass index, WC waist circumference, SBP systolic blood pressure, DBP diastolic blood pressure, WBC white blood cell, RBC red blood cell, FPG fasting plasma glucose, TC total cholesterol, TG triglyceride, HDL-C high-density lipoprotein cholesterol, LDL-C low-density lipoprotein cholesterol, ALT alanine aminotransferase, AST aspartate aminotransferase, GGT gamma-glutamyl transpeptidase, SUA serum uric acid, Scr serum creatinine, CMI cardiometabolic index, AIP atherogenic index of plasma, TyG triglyceride-glucose index, TyG-WC triglyceride-glucose × waist circumference, TyG-WHtR triglyceride-glucose × waist circumference/height

**Table S5.** Baseline characteristics of the study population according to triglyceride-glucose × waist circumference (TyG-WC) quartiles

| Variables | Q1 (405.31~613.52) (n=6342) | Q2 (613.52~686.18)  (n=6341) | Q3 (686.18~764.30)  (n=6342) | Q4 (764.30~1117.87) (n=6341) | *P* value |
| --- | --- | --- | --- | --- | --- |
| Sex, male, n (%) | 745 (11.75) | 2041 (32.19) | 3755 (59.21) | 5113 (80.63) | <0.001 |
| Age, years | 32.00 (28.00, 41.00) | 38.00 (31.00, 47.00) | 43.00 (33.00, 53.00) | 49.00 (37.00, 58.00) | <0.001 |
| Smoking status, n (%) | 17 (0.27) | 68 (1.07) | 165 (2.60) | 394 (6.21) | <0.001 |
| Drinking status, n (%) | 32 (0.50) | 91 (1.44) | 232 (3.66) | 431 (6.80) | <0.001 |
| BMI, kg/m^2^ | 20.34±1.88 | 22.33±1.98 | 23.90±2.10 | 25.99±2.44 | <0.001 |
| WC, cm | 69.48±4.46 | 77.47±3.85 | 84.04±4.07 | 92.20±5.62 | <0.001 |
| SBP, mmHg | 116.23±12.77 | 121.04±14.46 | 127.01±15.83 | 133.22±17.69 | <0.001 |
| DBP, mmHg | 70.56±8.84 | 72.69±9.53 | 75.93±10.30 | 80.02±10.98 | <0.001 |
| WBC, 10^9^/L | 5.60±1.36 | 5.76±1.37 | 5.97±1.41 | 6.37±1.53 | <0.001 |
| Neutrophils, 10^9^/L | 3.28±1.09 | 3.37±1.08 | 3.49±1.10 | 3.75±1.19 | <0.001 |
| Lymphocytes, 10^9^/L | 1.89±0.49 | 1.94±0.51 | 2.00±0.54 | 2.10±0.57 | <0.001 |
| RBC, 10^12^/L | 4.52±0.35 | 4.67±0.41 | 4.85±0.43 | 4.99±0.41 | <0.001 |
| Hemoglobin, g/L | 132.91±13.30 | 138.31±15.16 | 145.51±15.03 | 151.48±13.19 | <0.001 |
| Platelet, 10^9^/L | 242.85±51.53 | 243.63±53.95 | 235.70±53.38 | 230.92±52.31 | <0.001 |
| FPG, mmol/L | 5.16 (4.92, 5.40) | 5.31 (5.06, 5.58) | 5.44 (5.18, 5.75) | 5.66 (5.34, 6.11) | <0.001 |
| TC, mmol/L | 4.55±0.81 | 4.74±0.85 | 4.91±0.90 | 5.04±0.90 | <0.001 |
| TG, mmol/L | 0.80 (0.64, 1.02) | 1.03 (0.82, 1.34) | 1.26 (0.99, 1.62) | 1.71 (1.32, 2.27) | <0.001 |
| HDL-C, mmol/L | 1.59±0.30 | 1.46±0.30 | 1.35±0.28 | 1.24±0.26 | <0.001 |
| LDL-C, mmol/L | 2.27±0.61 | 2.50±0.64 | 2.71±0.68 | 2.79±0.69 | <0.001 |
| ALT, U/L | 13.00 (10.27, 16.84) | 15.00 (11.77, 20.00) | 18.00 (14.00, 24.12) | 21.27 (16.26, 28.72) | <0.001 |
| AST,U/L | 17.20 (15.05, 20.00) | 18.10 (15.74, 21.33) | 19.25 (16.69, 23.00) | 20.45 (17.62, 24.00) | <0.001 |
| GGT, U/L | 11.00 (9.00, 14.00) | 13.00 (10.00, 17.66) | 16.34 (12.27, 23.00) | 22.00 (15.94, 34.04) | <0.001 |
| SUA, μmol/L | 276.88±62.26 | 305.17±73.94 | 343.72±79.96 | 378.89±82.08 | <0.001 |
| Scr, μmol/L | 56.16 (50.93, 62.85) | 59.80 (52.92, 71.33) | 68.72 (57.36, 79.02) | 73.77 (64.77, 82.39) | <0.001 |
| CMI | 0.22 (0.17, 0.29) | 0.34 (0.26, 0.45) | 0.47 (0.36, 0.62) | 0.76 (0.55, 1.04) | <0.001 |
| AIP | -0.28±0.18 | -0.14±0.19 | -0.02±0.19 | 0.16±0.22 | <0.001 |
| TyG | 8.12±0.36 | 8.40±0.37 | 8.63±0.38 | 9.01±0.44 | <0.001 |
| TyG-BMI | 165.09±15.94 | 187.45±16.62 | 205.90±17.67 | 234.05±22.77 | <0.001 |
| TyG-WHtR | 3.44±0.24 | 3.91±0.21 | 4.28±0.24 | 4.83±0.37 | <0.001 |
| Hypertension, n (%) | 282 (4.45) | 643 (10.14) | 1332 (21.00) | 2285 (36.04) | <0.001 |
| Diabetes, n (%) | 18 (0.28) | 63 (0.99) | 199 (3.14) | 729 (11.50) | <0.001 |
| Dyslipidemia , n (%) | 316 (4.98) | 640 (10.09) | 1246 (19.65) | 2587 (40.80) | <0.001 |

The data are presented as the means ± SDs, n (%), or medians (quartile 1, quartile 3).

Abbreviations: MASLD metabolic dysfunction-associated steatotic liver disease, BMI body mass index, WC waist circumference, SBP systolic blood pressure, DBP diastolic blood pressure, WBC white blood cell, RBC red blood cell, FPG fasting plasma glucose, TC total cholesterol, TG triglyceride, HDL-C high-density lipoprotein cholesterol, LDL-C low-density lipoprotein cholesterol, ALT alanine aminotransferase, AST aspartate aminotransferase, GGT gamma-glutamyl transpeptidase, SUA serum uric acid, Scr serum creatinine, CMI cardiometabolic index, AIP atherogenic index of plasma, TyG triglyceride-glucose index, TyG-BMI triglyceride-glucose × body mass index, TyG-WHtR triglyceride-glucose × waist circumference/height

**Table S6.** Baseline characteristics of the study population according to triglyceride-glucose × waist circumference/height (TyG-WHtR) quartiles

| Variables | Q1 (405.31~613.52) (n=6342) | Q2 (613.52~686.18)  (n=6341) | Q3 (686.18~764.30)  (n=6342) | Q4 (764.30~1117.87) (n=6341) | *P* value |
| --- | --- | --- | --- | --- | --- |
| Sex, male, n (%) | 1438 (22.67) | 2503 (39.47) | 3491 (55.05) | 4222 (66.58) | <0.001 |
| Age, years | 32.00 (27.00, 39.00) | 37.00 (30.00, 47.00) | 43.00 (33.00, 52.00) | 50.00 (40.00, 60.00) | <0.001 |
| Smoking status, n (%) | 32 (0.50) | 76 (1.20) | 174 (2.74) | 362 (5.71) | <0.001 |
| Drinking status, n (%) | 23 (0.36) | 85 (1.34) | 182 (2.87) | 496 (7.82) | <0.001 |
| BMI, kg/m^2^ | 20.31±1.88 | 22.33±1.93 | 23.90±2.04 | 26.02±2.46 | <0.001 |
| WC, cm | 70.28±5.34 | 77.95±5.24 | 83.79±5.48 | 91.16±6.43 | <0.001 |
| SBP, mmHg | 116.76±12.71 | 120.97±14.20 | 125.88±15.31 | 133.90±18.41 | <0.001 |
| DBP, mmHg | 70.81±8.83 | 72.82±9.57 | 75.77±10.23 | 79.79±11.26 | <0.001 |
| WBC, 10^9^/L | 5.59±1.35 | 5.80±1.38 | 5.99±1.42 | 6.32±1.54 | <0.001 |
| Neutrophils, 10^9^/L | 3.26±1.08 | 3.39±1.09 | 3.51±1.11 | 3.74±1.19 | <0.001 |
| Lymphocytes, 10^9^/L | 1.90±0.49 | 1.96±0.51 | 2.00±0.54 | 2.07±0.57 | <0.001 |
| RBC, 10^12^/L | 4.59±0.39 | 4.71±0.43 | 4.83±0.44 | 4.90±0.44 | <0.001 |
| Hemoglobin, g/L | 135.58±14.66 | 139.86±15.61 | 144.50±15.45 | 148.26±14.70 | <0.001 |
| Platelet, 10^9^/L | 238.89±50.35 | 241.51±52.87 | 238.69±54.07 | 234.01±54.58 | <0.001 |
| FPG, mmol/L | 5.17 (4.93, 5.40) | 5.31 (5.06, 5.57) | 5.44 (5.18, 5.74) | 5.67 (5.34, 6.13) | <0.001 |
| TC, mmol/L | 4.51±0.79 | 4.71±0.83 | 4.91±0.87 | 5.11±0.92 | <0.001 |
| TG, mmol/L | 0.79 (0.63, 1.01) | 1.03 (0.82, 1.32) | 1.28 (1.00, 1.65) | 1.71 (1.32, 2.28) | <0.001 |
| HDL-C, mmol/L | 1.58±0.31 | 1.45±0.30 | 1.36±0.29 | 1.27±0.28 | <0.001 |
| LDL-C, mmol/L | 2.26±0.60 | 2.50±0.63 | 2.69±0.67 | 2.81±0.70 | <0.001 |
| ALT, U/L | 13.00 (10.45, 17.00) | 15.60 (12.00, 21.00) | 17.94 (13.66, 24.01) | 20.61 (15.69, 28.00) | <0.001 |
| AST,U/L | 17.35 (15.16, 20.00) | 18.16 (15.85, 21.32) | 19.14 (16.61, 22.88) | 20.44 (17.53, 24.09) | <0.001 |
| GGT, U/L | 11.05 (9.00, 14.65) | 13.09 (10.14, 18.34) | 16.21 (12.00, 23.37) | 21.00 (14.87, 33.00) | <0.001 |
| SUA, μmol/L | 287.88±70.45 | 313.40±77.97 | 338.62±82.65 | 364.70±85.36 | <0.001 |
| Scr, μmol/L | 57.86 (51.80, 66.68) | 61.64 (53.49, 74.12) | 67.00 (56.00, 78.16) | 70.41 (59.51, 80.11) | <0.001 |
| CMI | 0.22 (0.17, 0.28) | 0.34 (0.26, 0.44) | 0.48 (0.36, 0.64) | 0.75 (0.54, 1.04) | <0.001 |
| AIP | -0.28±0.18 | -0.13±0.19 | -0.01±0.20 | 0.15±0.22 | <0.001 |
| TyG | 8.11±0.35 | 8.40±0.36 | 8.64±0.37 | 9.02±0.44 | <0.001 |
| TyG-BMI | 164.54±15.54 | 187.31±15.58 | 206.25±16.86 | 234.38±22.89 | <0.001 |
| TyG-WC | 569.40±43.39 | 653.63±38.65 | 722.98±42.34 | 821.41±63.49 | <0.001 |
| Hypertension, n (%) | 273 (4.30) | 655 (10.33) | 1164 (18.35) | 2450 (38.64) | <0.001 |
| Diabetes, n (%) | 18 (0.28) | 60 (0.95) | 162 (2.55) | 769 (12.13) | <0.001 |
| Dyslipidemia , n (%) | 301 (4.75) | 634 (10.00) | 1250 (19.71) | 2604 (41.07) | <0.001 |

The data are presented as the means ± SDs, n (%), or medians (quartile 1, quartile 3).

Abbreviations: MASLD metabolic dysfunction-associated steatotic liver disease, BMI body mass index, WC waist circumference, SBP systolic blood pressure, DBP diastolic blood pressure, WBC white blood cell, RBC red blood cell, FPG fasting plasma glucose, TC total cholesterol, TG triglyceride, HDL-C high-density lipoprotein cholesterol, LDL-C low-density lipoprotein cholesterol, ALT alanine aminotransferase, AST aspartate aminotransferase, GGT gamma-glutamyl transpeptidase, SUA serum uric acid, Scr serum creatinine, CMI cardiometabolic index, AIP atherogenic index of plasma, TyG triglyceride-glucose index, TyG-BMI triglyceride-glucose × body mass index, TyG-WC triglyceride-glucose × waist circumference

**Table S7.** Comparison of the incidence rate and incidence density of MASLD in different groups

| Categories | Event/Total | Incidence rate (%) | Total person-years | Incidence density (/1000 person-years) | $x^{2}$ value | *P* value |
| --- | --- | --- | --- | --- | --- | --- |
| CMI |  |  |  |  | 2426.66 | <0.001 |
| Q1 | 399/6342 | 6.29 | 23124.92 | 17.25 |  |  |
| Q2 | 905/6341 | 14.27 | 21833.08 | 41.45 |  |  |
| Q3 | 1525/6342 | 24.05 | 19903.25 | 76.62 |  |  |
| Q4 | 2549/6341 | 40.20 | 17583.33 | 144.97 |  |  |
| AIP |  |  |  |  | 2036.25 | <0.001 |
| Q1 | 472/6344 | 7.44 | 23019.83 | 20.50 |  |  |
| Q2 | 970/6340 | 15.30 | 21426.25 | 45.27 |  |  |
| Q3 | 1480/6343 | 23.33 | 20005.75 | 73.98 |  |  |
| Q4 | 2456/6339 | 38.74 | 17992.75 | 136.50 |  |  |
| TyG |  |  |  |  | 1503.13 | <0.001 |
| Q1 | 580/6342 | 9.15 | 23340.67 | 24.85 |  |  |
| Q2 | 1045/6341 | 16.48 | 21649.92 | 48.27 |  |  |
| Q3 | 1457/6342 | 22.97 | 19534.58 | 74.59 |  |  |
| Q4 | 2296/6341 | 36.21 | 17919.42 | 128.13 |  |  |
| TyG-BMI |  |  |  |  | 3351.53 | <0.001 |
| Q1 | 240/6342 | 3.78 | 23625.42 | 10.16 |  |  |
| Q2 | 805/6341 | 12.70 | 22107.50 | 36.41 |  |  |
| Q3 | 1580/6342 | 24.91 | 19939.83 | 79.24 |  |  |
| Q4 | 2753/6341 | 43.42 | 16771.83 | 164.14 |  |  |
| TyG-WC |  |  |  |  | 2881.10 | <0.001 |
| Q1 | 283/6342 | 4.46 | 23866.92 | 11.86 |  |  |
| Q2 | 893/6341 | 14.08 | 22170.50 | 40.28 |  |  |
| Q3 | 1564/6342 | 24.66 | 19897.58 | 78.60 |  |  |
| Q4 | 2638/6341 | 41.60 | 16509.58 | 159.79 |  |  |
| TyG-WHtR |  |  |  |  | 2596.70 | <0.001 |
| Q1 | 280/6342 | 4.42 | 23497.92 | 11.92 |  |  |
| Q2 | 964/6341 | 15.20 | 22291.25 | 43.25 |  |  |
| Q3 | 1604/6342 | 25.29 | 19888.00 | 80.65 |  |  |
| Q4 | 2530/6341 | 39.90 | 16767.42 | 150.89 |  |  |
| Sex |  |  |  |  | 901.69 | <0.001 |
| Male | 3445/11654 | 29.56 | 34078.50 | 101.09 |  |  |
| Female | 1933/13712 | 14.10 | 48366.08 | 39.97 |  |  |
| Age (years) |  |  |  |  | 156.15 | <0.001 |
| 18-44 | 2877/15349 | 18.74 | 52379.25 | 54.93 |  |  |
| 45-59 | 1888/7273 | 25.96 | 22566.58 | 83.66 |  |  |
| ≥ 60 | 613/2744 | 22.34 | 7498.75 | 81.75 |  |  |
| Total | 5378/25366 | 21.20 | 82444.58 | 65.23 |  |  |

Abbreviations: MASLD metabolic dysfunction-associated steatotic liver disease, CMI cardiometabolic index, AIP atherogenic index of plasma, TyG triglyceride-glucose index, TyG-BMI triglyceride-glucose × body mass index, TyG-WC triglyceride-glucose × waist circumference, TyG-WHtR triglyceride-glucose × waist circumference/height

**Table S8.** Association between cardiometabolic indices and MASLD components

| Variables | CMI | AIP | TyG | TyG-BMI | TyG-WC | TyG-WHtR |
| --- | --- | --- | --- | --- | --- | --- |
| Obesity |  |  |  |  |  |  |
| Per SD increase | 1.28 (1.26, 1.31)*** | 1.67 (1.61, 1.73)*** | 1.54 (1.49, 1.60)*** | 2.36 (2.29, 2.44)*** | 2.48 (2.39, 2.57)*** | 2.36 (2.28, 2.44)*** |
| Quartiles |  |  |  |  |  |  |
| Q1 | Reference | Reference | Reference | Reference | Reference | Reference |
| Q2 | 2.29 (2.01, 2.60)*** | 2.03 (1.80, 2.28)*** | 1.74 (1.56, 1.94)*** | 4.15 (3.45, 5.00)*** | 3.88 (3.32, 4.55)*** | 3.89 (3.32, 4.54)*** |
| Q3 | 3.94 (3.49, 4.45)*** | 3.03 (2.70, 3.39)*** | 2.49 (2.24, 2.77)*** | 10.69 (8.97, 12.74)*** | 8.10 (6.95, 9.45)*** | 7.84 (6.75, 9.12)*** |
| Q4 | 6.45 (5.70, 7.31)*** | 4.57 (4.07, 5.13)*** | 3.44 (3.09, 3.83)*** | 22.92 (19.25, 27.30)*** | 17.59 (15.04, 20.58)*** | 15.37 (13.20, 17.91)*** |
| *P*_trend_ | <0.001 | <0.001 | <0.001 | <0.001 | <0.001 | <0.001 |
| Raised FPG or DM |  |  |  |  |  |  |
| Per SD increase | 1.28 (1.25, 1.31)*** | 1.67 (1.60, 1.74)*** | 1.70 (1.63, 1.78)*** | 2.16 (2.08, 2.24)*** | 2.35 (2.25, 2.46)*** | 2.21 (2.12, 2.30)*** |
| Quartiles |  |  |  |  |  |  |
| Q1 | Reference | Reference | Reference | Reference | Reference | Reference |
| Q2 | 2.27 (1.93, 2.68)*** | 2.03 (1.74, 2.36)*** | 1.81 (1.56, 2.10)*** | 3.13 (2.55, 3.83)*** | 3.23 (2.66, 3.94)*** | 3.33 (2.74, 4.05)*** |
| Q3 | 3.88 (3.32, 4.53)*** | 3.04 (2.63, 3.50)*** | 2.88 (2.50, 3.32)*** | 6.37 (5.25, 7.72)*** | 6.55 (5.42, 7.92)*** | 6.33 (5.24, 7.64)*** |
| Q4 | 6.32 (5.39, 7.40)*** | 4.64 (4.01, 5.37)*** | 4.48 (3.88, 5.16)*** | 13.07 (10.79, 15.82)*** | 13.77 (11.35, 16.70)*** | 12.54 (10.38, 15.16)*** |
| *P*_trend_ | <0.001 | <0.001 | <0.001 | <0.001 | <0.001 | <0.001 |
| Raised BP |  |  |  |  |  |  |
| Per SD increase | 1.25 (1.22, 1.28)*** | 1.61 (1.54, 1.68)*** | 1.54 (1.47, 1.61)*** | 2.16 (2.08, 2.25)*** | 2.28 (2.18, 2.39)*** | 2.16 (2.07, 2.26)*** |
| Quartiles |  |  |  |  |  |  |
| Q1 | Reference | Reference | Reference | Reference | Reference | Reference |
| Q2 | 2.15 (1.81, 2.56)*** | 1.90 (1.63, 2.23)*** | 1.66 (1.43, 1.93)*** | 3.65 (2.87, 4.63)*** | 3.74 (3.00, 4.67)*** | 3.68 (2.96, 4.59)*** |
| Q3 | 3.88 (3.29, 4.57)*** | 2.90 (2.50, 3.36)*** | 2.43 (2.11, 2.80)*** | 8.13 (6.48, 10.21)*** | 7.30 (5.89, 9.05)*** | 7.23 (5.85, 8.93)*** |
| Q4 | 5.89 (4.99, 6.96)*** | 4.18 (3.60, 4.86)*** | 3.48 (3.01, 4.02)*** | 17.11 (13.65, 21.45)*** | 15.53 (12.50, 19.30)*** | 13.85 (11.20, 17.13)*** |
| *P*_trend_ | <0.001 | <0.001 | <0.001 | <0.001 | <0.001 | <0.001 |
| Raised TG |  |  |  |  |  |  |
| Per SD increase | 1.34 (1.31, 1.36)*** | 2.21 (2.12, 2.31)*** | 2.09 (2.00, 2.18)*** | 2.23 (2.15, 2.31)*** | 2.48 (2.37, 2.59)*** | 2.38 (2.28, 2.48)*** |
| Quartiles |  |  |  |  |  |  |
| Q1 | Reference | Reference | Reference | Reference | Reference | Reference |
| Q2 | 3.19 (2.54, 4.00)*** | 3.28 (2.64, 4.06)*** | 2.79 (2.30, 3.39)*** | 3.74 (3.00, 4.66)*** | 3.83 (3.10, 4.74)*** | 3.99 (3.22, 4.94)*** |
| Q3 | 7.90 (6.39, 9.77)*** | 6.64 (5.43, 8.13)*** | 5.71 (4.76, 6.85)*** | 7.95 (6.44, 9.81)*** | 8.32 (6.78, 10.20)*** | 8.49 (6.92, 10.43)*** |
| Q4 | 18.46 (14.94, 22.81)*** | 14.60 (11.95, 17.84)*** | 10.87 (9.07, 13.01)*** | 17.69 (14.36, 21.79)*** | 19.15 (15.57, 23.56)*** | 17.98 (14.63, 22.10)*** |
| *P*_trend_ | <0.001 | <0.001 | <0.001 | <0.001 | <0.001 | <0.001 |
| Reduced HDL-C |  |  |  |  |  |  |
| Per SD increase | 1.34 (1.31, 1.37)*** | 2.07 (1.99, 2.16)*** | 1.72 (1.65, 1.80)*** | 2.29 (2.21, 2.38)*** | 2.48 (2.37, 2.59)*** | 2.36 (2.26, 2.46)*** |
| Quartiles |  |  |  |  |  |  |
| Q1 | Reference | Reference | Reference | Reference | Reference | Reference |
| Q2 | 2.98 (2.48, 3.59)*** | 2.91 (2.44, 3.46)*** | 2.09 (1.82, 2.41)*** | 3.83 (3.15, 4.65)*** | 3.46 (2.90, 4.13)*** | 3.54 (2.98, 4.22)*** |
| Q3 | 6.52 (5.49, 7.76)*** | 5.35 (4.54, 6.30)*** | 3.23 (2.82, 3.70)*** | 7.97 (6.61, 9.61)*** | 7.46 (6.29, 8.85)*** | 6.83 (5.76, 8.08)*** |
| Q4 | 13.01 (10.93, 15.49)*** | 9.82 (8.33, 11.58)*** | 5.02 (4.38, 5.76)*** | 17.67 (14.67, 21.27)*** | 15.79 (13.25, 18.81)*** | 13.74 (11.58, 16.30)*** |
| *P*_trend_ | <0.001 | <0.001 | <0.001 | <0.001 | <0.001 | <0.001 |

The HR (95% CI) was adjusted for age, sex, smoking status, drinking status, hypertension, diabetes and dyslipidemia.

Abbreviations: MASLD metabolic dysfunction-associated steatotic liver disease, FPG fasting plasma glucose, DM diabetes mellitus, BP blood pressure, TG triglyceride, HDL-C high-density lipoprotein cholesterol, CMI cardiometabolic index, AIP atherogenic index of plasma, TyG triglyceride-glucose index, TyG-BMI triglyceride-glucose × body mass index, TyG-WC triglyceride-glucose × waist circumference, TyG-WHtR triglyceride-glucose × waist circumference/height

**P* < 0.05, ***P* < 0.01, and ****P* < 0.001

**Table S9.** Baseline characteristics of the study population with and without liver fibrosis

| Variables | Overall (n=1,646) | MASLD (n=1,390) | Fibrosis (n=256) | *P* value |
| --- | --- | --- | --- | --- |
| Sex, male, n (%) | 1,112 (67.56) | 914 (65.76) | 198 (77.34) | <0.001 |
| Age, years | 45.92±12.39 | 44.20±12.12 | 55.26±9.29 | <0.001 |
| Smoking status, n (%) | 69 (4.19) | 47 (3.38) | 22 (8.59) | <0.001 |
| Drinking status, n (%) | 61 (3.71) | 43 (3.09) | 18 (7.03) | 0.004 |
| BMI, kg/m^2^ | 25.95±2.62 | 25.85±2.61 | 26.46±2.65 | 0.001 |
| WC, cm | 89.62±7.71 | 89.23±7.66 | 91.73±7.65 | <0.001 |
| SBP, mmHg | 109.00 (102.00, 118.00) | 108.00 (101.00, 117.00) | 113.50 (105.00, 126.00) | <0.001 |
| DBP, mmHg | 66.00 (64.00, 68.00) | 65.95 (64.00, 68.00) | 67.00 (65.00, 70.00) | <0.001 |
| WBC, 10^9^/L | 6.25±1.47 | 6.27±1.48 | 6.12±1.44 | 0.143 |
| Neutrophils, 10^9^/L | 3.68±1.14 | 3.68±1.16 | 3.66±1.08 | 0.772 |
| Lymphocytes, 10^9^/L | 2.08±0.54 | 2.09±0.54 | 1.99±0.56 | 0.004 |
| RBC, 10^12^/L | 4.94±0.41 | 4.94±0.41 | 4.93±0.39 | 0.660 |
| Hemoglobin, g/L | 148.70±14.43 | 148.32±14.59 | 150.77±13.41 | 0.013 |
| Platelet, 10^9^/L | 241.17±54.61 | 248.71±53.04 | 200.22±43.80 | <0.001 |
| FPG, mmol/L | 5.72 (5.21, 6.00) | 5.64 (5.15, 6.00) | 6.18 (5.70, 7.82) | <0.001 |
| TC, mmol/L | 5.15±0.96 | 5.17±0.94 | 5.07±1.04 | 0.123 |
| TG, mmol/L | 1.97 (1.15, 2.36) | 1.96 (1.14, 2.34) | 2.00 (1.25, 2.46) | 0.216 |
| HDL-C, mmol/L | 1.19±0.31 | 1.19±0.31 | 1.23±0.34 | 0.039 |
| LDL-C, mmol/L | 2.97±0.78 | 3.00±0.76 | 2.80±0.82 | <0.001 |
| ALT, U/L | 24.25 (18.00, 34.12) | 24.68 (18.05, 35.00) | 22.95 (16.88, 32.00) | 0.021 |
| AST,U/L | 21.45 (18.43, 26.00) | 21.39 (18.45, 26.00) | 21.84 (18.00, 27.00) | 0.481 |
| GGT, U/L | 24.00 (17.00, 37.00) | 24.00 (16.71, 36.28) | 25.29 (18.00, 40.21) | 0.047 |
| SUA, μmol/L | 364.95±85.10 | 363.05±85.44 | 375.33±82.63 | 0.035 |
| Scr, μmol/L | 69.67±14.61 | 69.41±14.55 | 71.12±14.87 | 0.086 |
| CMI | 0.83 (0.52, 1.15) | 0.82 (0.52, 1.14) | 0.86 (0.52, 1.20) | 0.297 |
| AIP | 0.18±0.26 | 0.18±0.26 | 0.18±0.27 | 0.910 |
| TyG | 9.00±0.52 | 8.97±0.49 | 9.19±0.59 | <0.001 |
| TyG-BMI | 233.68±27.39 | 231.90±26.50 | 243.38±30.06 | <0.001 |
| TyG-WC | 807.49±88.01 | 800.78±84.72 | 843.94±96.43 | <0.001 |
| TyG-WHtR | 4.73±0.48 | 4.69±0.46 | 4.96±0.53 | <0.001 |
| Hypertension, n (%) | 215 (13.06) | 137 (9.86) | 78 (30.47) | <0.001 |
| Diabetes, n (%) | 172 (10.45) | 68 (4.89) | 104 (40.62) | <0.001 |
| Dyslipidemia , n (%) | 660 (40.10) | 549 (39.50) | 111 (43.36) | 0.276 |

The data are presented as the means ± SDs, n (%), or medians (quartile 1, quartile 3).

Abbreviations: MASLD metabolic dysfunction-associated steatotic liver disease, BMI body mass index, WC waist circumference, SBP systolic blood pressure, DBP diastolic blood pressure, WBC white blood cell, RBC red blood cell, FPG fasting plasma glucose, TC total cholesterol, TG triglyceride, HDL-C high-density lipoprotein cholesterol, LDL-C low-density lipoprotein cholesterol, ALT alanine aminotransferase, AST aspartate aminotransferase, GGT gamma-glutamyl transpeptidase, SUA serum uric acid, Scr serum creatinine, CMI cardiometabolic index, AIP atherogenic index of plasma, TyG triglyceride-glucose index, TyG-BMI triglyceride-glucose × body mass index, TyG-WC triglyceride-glucose × waist circumference, TyG-WHtR triglyceride-glucose × waist circumference/height

**Table S10.** Association between cardiometabolic indices and non-invasive fibrosis score

| Variables | Model 1  *HR* (95%*CI*) | *P* value | Model 2  *HR* (95%*CI*) | *P* value | Model 3  *HR* (95%*CI*) | *P* value |
| --- | --- | --- | --- | --- | --- | --- |
| MASLD with MAF-5 ≥ 1 |  |  |  |  |  |  |
| CMI |  |  |  |  |  |  |
| Per SD increase | 1.25 (1.11, 1.41) | <0.001 | 1.24 (1.10, 1.40) | <0.001 | 1.11 (0.96, 1.29) | 0.158 |
| Quartiles |  |  |  |  |  |  |
| Q1 | Reference |  | Reference |  | Reference |  |
| Q2 | 1.58 (0.93, 2.70) | 0.091 | 1.56 (0.92, 2.66) | 0.102 | 1.53 (0.90, 2.62) | 0.117 |
| Q3 | 1.91 (1.14, 3.20) | 0.014 | 1.98 (1.19, 3.32) | 0.009 | 1.71 (1.01, 2.89) | 0.045 |
| Q4 | 2.50 (1.53, 4.09) | <0.001 | 2.36 (1.44, 3.87) | 0.001 | 1.88 (1.01, 3.49) | 0.045 |
| *P*_trend_ |  | <0.001 |  | <0.001 |  | 0.035 |
| AIP |  |  |  |  |  |  |
| Per SD increase | 1.22 (1.04, 1.43) | 0.015 | 1.22 (1.04, 1.43) | 0.014 | 1.06 (0.87, 1.28) | 0.582 |
| Quartiles |  |  |  |  |  |  |
| Q1 | Reference |  | Reference |  | Reference |  |
| Q2 | 1.07 (0.63, 1.79) | 0.812 | 1.05 (0.62, 1.76) | 0.863 | 1.03 (0.61, 1.74) | 0.910 |
| Q3 | 1.61 (1.02, 2.54) | 0.040 | 1.71 (1.09, 2.70) | 0.021 | 1.47 (0.93, 2.35) | 0.102 |
| Q4 | 1.69 (1.08, 2.64) | 0.022 | 1.68 (1.07, 2.64) | 0.024 | 1.12 (0.63, 2.00) | 0.705 |
| *P*_trend_ |  | 0.007 |  | 0.006 |  | 0.312 |
| TyG |  |  |  |  |  |  |
| Per SD increase | 1.53 (1.33, 1.75) | <0.001 | 1.46 (1.27, 1.68) | <0.001 | 1.12 (0.92, 1.36) | 0.257 |
| Quartiles |  |  |  |  |  |  |
| Q1 | Reference |  | Reference |  | Reference |  |
| Q2 | 1.45 (0.82, 2.55) | 0.201 | 1.47 (0.84, 2.59) | 0.181 | 1.30 (0.74, 2.31) | 0.363 |
| Q3 | 1.97 (1.14, 3.38) | 0.015 | 1.89 (1.10, 3.25) | 0.022 | 1.47 (0.83, 2.60) | 0.182 |
| Q4 | 3.72 (2.26, 6.10) | <0.001 | 3.27 (1.99, 5.37) | <0.001 | 1.72 (0.92, 3.20) | 0.088 |
| *P*_trend_ |  | <0.001 |  | <0.001 |  | 0.088 |
| TyG-BMI |  |  |  |  |  |  |
| Per SD increase | 1.83 (1.58, 2.11) | <0.001 | 1.89 (1.63, 2.19) | <0.001 | 1.65 (1.40, 1.93) | <0.001 |
| Quartiles |  |  |  |  |  |  |
| Q1 | Reference |  | Reference |  | Reference |  |
| Q2 | 2.01 (1.02, 3.95) | 0.042 | 1.87 (0.95, 3.67) | 0.070 | 1.77 (0.89, 3.49) | 0.102 |
| Q3 | 3.08 (1.65, 5.77) | <0.001 | 2.74 (1.46, 5.14) | 0.002 | 2.33 (1.23, 4.42) | 0.010 |
| Q4 | 5.93 (3.28, 10.72) | <0.001 | 5.98 (3.30, 10.84) | <0.001 | 4.47 (2.40, 8.32) | <0.001 |
| *P*_trend_ |  | <0.001 |  | <0.001 |  | <0.001 |
| TyG-WC |  |  |  |  |  |  |
| Per SD increase | 2.21 (1.90, 2.56) | <0.001 | 2.13 (1.83, 2.49) | <0.001 | 1.86 (1.56, 2.21) | <0.001 |
| Quartiles |  |  |  |  |  |  |
| Q1 | Reference |  | Reference |  | Reference |  |
| Q2 | 1.75 (0.88, 3.48) | 0.108 | 1.55 (0.77, 3.12) | 0.215 | 1.51 (0.75, 3.05) | 0.248 |
| Q3 | 2.59 (1.34, 4.98) | 0.004 | 2.17 (1.11, 4.25) | 0.024 | 1.94 (0.98, 3.87) | 0.058 |
| Q4 | 7.89 (1.39, 14.20) | <0.001 | 6.67 (3.60, 12.36) | <0.001 | 5.09 (2.63, 9.86) | <0.001 |
| *P*_trend_ |  | <0.001 |  | <0.001 |  | <0.001 |
| TyG-WHtR |  |  |  |  |  |  |
| Per SD increase | 1.99 (1.75, 2.27) | <0.001 | 1.89 (1.65, 2.16) | <0.001 | 1.64 (1.40, 1.93) | <0.001 |
| Quartiles |  |  |  |  |  |  |
| Q1 | Reference |  | Reference |  | Reference |  |
| Q2 | 2.17 (1.01, 4.68) | 0.047 | 1.90 (0.88, 4.10) | 0.101 | 1.79 (0.82, 3.87) | 0.141 |
| Q3 | 5.21 (2.62, 10.38) | <0.001 | 4.23 (2.12, 8.47) | <0.001 | 3.60 (1.76, 7.33) | <0.001 |
| Q4 | 10.25 (5.28, 19.92) | <0.001 | 8.15 (4.16, 15.97) | <0.001 | 6.30 (3.08, 12.88) | <0.001 |
| *P*_trend_ |  | <0.001 |  | <0.001 |  | <0.001 |

We excluded 2,721 individuals who lost follow-up and 1,494 individuals without required data from 5,378 MASLDs. In total, 1,163 subjects diagnosed with MASLD were examined to evaluate the relationship between cardiometabolic indices and subsequent liver fibrosis.

Model 1: Unadjusted

Model 2: Adjusted for age and sex

Model 3: Adjusted for age, sex, smoking status, drinking status, hypertension, diabetes and dyslipidemia

HR hazard ratio, CI confidence interval, MASLD metabolic dysfunction-associated steatotic liver disease, CMI cardiometabolic index, AIP atherogenic index of plasma, TyG triglyceride-glucose index, TyG-BMI triglyceride-glucose × body mass index, TyG-WC triglyceride-glucose × waist circumference, TyG-WHtR triglyceride-glucose × waist circumference/height

**Table S11.** Association between cardiometabolic index (CMI) and MASLD stratified by age, sex, BMI, hypertension, diabetes and dyslipidemia status

| Subgroup | Q1  *HR* (95%CI) | Q2  *HR* (95%CI) | Q3  *HR* (95%CI) | Q4  *HR* (95%CI) | *P* _interaction_ |
| --- | --- | --- | --- | --- | --- |
| Age |  |  |  |  | <0.001 |
| 18-44 | Ref | 2.32 (1.95, 2.77)*** | 4.40 (3.74, 5.19)*** | 6.94 (5.88, 8.20)*** |  |
| 45-59 | Ref | 1.79 (1.50, 2.13)*** | 2.70 (2.29, 3.19)*** | 4.19 (3.53, 4.98)*** |  |
| ≥ 60 | Ref | 1.72 (1.29, 2.29)*** | 2.52 (1.92, 3.32)*** | 4.01 (3.04, 5.28)*** |  |
| Sex |  |  |  |  | <0.001 |
| Male | Ref | 1.90 (1.68, 2.14)*** | 2.69 (2.39, 3.02)*** | 3.86 (3.40, 4.37)*** |  |
| Female | Ref | 2.26 (1.84, 2.78)*** | 4.22 (3.48, 5.12)*** | 7.88 (6.53, 9.51)*** |  |
| BMI |  |  |  |  | <0.001 |
| < 24 | Ref | 2.14 (1.75, 2.62)*** | 3.70 (3.06, 4.47)*** | 6.24 (5.17, 7.54)*** |  |
| ≥ 24 | Ref | 1.53 (1.37, 1.71)*** | 1.85 (1.67, 2.06)*** | 2.41 (2.14, 2.71)*** |  |
| Hypertension |  |  |  |  | <0.001 |
| No | Ref | 2.24 (1.95, 2.58)*** | 3.92 (3.43, 4.48)*** | 6.87 (6.01, 7.85)*** |  |
| Yes | Ref | 1.71 (1.42, 2.06)*** | 2.20 (1.84, 2.64)*** | 3.31 (2.73, 4.03)*** |  |
| Diabetes |  |  |  |  | 0.974 |
| No | Ref | 2.22 (1.96, 2.51)*** | 3.83 (3.41, 4.30)*** | 6.20 (5.51, 6.98)*** |  |
| Yes | Ref | 2.13 (1.44, 3.16)*** | 2.48 (1.69, 3.64)*** | 3.78 (2.51, 5.69)*** |  |
| Dyslipidemia |  |  |  |  | 0.553 |
| No | Ref | 2.06 (1.79, 2.37)*** | 3.55 (3.11, 4.04)*** | 5.79 (5.09, 6.58)*** |  |
| Yes | Ref | 1.78 (1.50, 2.10)*** | 2.38 (2.02, 2.81)*** | 3.13 (2.66, 3.68)*** |  |

Adjusted for age, sex, smoking status, drinking status, hypertension, diabetes and dyslipidemia. In each subgroup, variables other than subgroup variables were adjusted

**P* < 0.05, ***P* < 0.01, and ****P* < 0.001

**Table S12.** Association between atherogenic index of plasma (AIP) and MASLD stratified by age, sex, BMI, hypertension, diabetes and dyslipidemia status

| Subgroup | Q1  *HR* (95%CI) | Q2  *HR* (95%CI) | Q3  *HR* (95%CI) | Q4  *HR* (95%CI) | *P* _interaction_ |
| --- | --- | --- | --- | --- | --- |
| Age |  |  |  |  | <0.001 |
| 18-44 | Ref | 2.27 (1.93, 2.67)*** | 3.58 (3.07, 4.18)*** | 5.35 (4.58, 6.25)*** |  |
| 45-59 | Ref | 1.50 (1.27, 1.78)*** | 2.16 (1.85, 2.53)*** | 3.12 (2.65, 3.68)*** |  |
| ≥ 60 | Ref | 1.69 (1.29, 2.23)*** | 2.18 (1.68, 2.85)*** | 3.22 (2.46, 4.22)*** |  |
| Sex |  |  |  |  | <0.001 |
| Male | Ref | 1.73 (1.54, 1.95)*** | 2.34 (2.09, 2.62)*** | 2.99 (2.64, 3.38)*** |  |
| Female | Ref | 1.80 (1.50, 2.15)*** | 2.87 (2.42, 3.40)*** | 5.17 (4.39, 6.10)*** |  |
| BMI |  |  |  |  | <0.001 |
| < 24 | Ref | 1.73 (1.43, 2.09)*** | 2.81 (2.36, 3.35)*** | 4.80 (4.05, 5.71)*** |  |
| ≥ 24 | Ref | 1.51 (1.35, 1.68)*** | 1.78 (1.60, 1.98)*** | 2.23 (1.99, 2.51)*** |  |
| Hypertension |  |  |  |  | <0.001 |
| No | Ref | 2.05 (1.79, 2.33)*** | 3.24 (2.87, 3.67)*** | 5.06 (4.46, 5.73)*** |  |
| Yes | Ref | 1.58 (1.32, 1.90)*** | 1.96 (1.64, 2.34)*** | 2.71 (2.23, 3.28)*** |  |
| Diabetes |  |  |  |  | 0.582 |
| No | Ref | 1.96 (1.75, 2.20)*** | 3.02 (2.71, 3.37)*** | 4.52 (4.05, 5.05)*** |  |
| Yes | Ref | 1.84 (1.26, 2.71)** | 2.06 (1.41, 3.01)*** | 3.61 (2.40, 5.41)*** |  |
| Dyslipidemia |  |  |  |  | 0.422 |
| No | Ref | 1.83 (1.61, 2.08)*** | 2.79 (2.47, 3.15)*** | 4.21 (3.74, 4.73)*** |  |
| Yes | Ref | 1.80 (1.53, 2.12)*** | 2.17 (1.85, 2.56)*** | 2.77 (2.36, 3.24)*** |  |

Adjusted for age, sex, smoking status, drinking status, hypertension, diabetes and dyslipidemia. In each subgroup, variables other than subgroup variables were adjusted

**P* < 0.05, ***P* < 0.01, and ****P* < 0.001

**Table S13.** Association between triglyceride-glucose index (TyG) and MASLD stratified by age, sex, BMI, hypertension, diabetes and dyslipidemia status

| Subgroup | Q1  *HR* (95%CI) | Q2  *HR* (95%CI) | Q3  *HR* (95%CI) | Q4  *HR* (95%CI) | *P* _interaction_ |
| --- | --- | --- | --- | --- | --- |
| Age |  |  |  |  | 0.001 |
| 18-44 | Ref | 1.77 (1.53, 2.04)*** | 2.75 (2.40, 3.16)*** | 3.71 (3.23, 4.26)*** |  |
| 45-59 | Ref | 1.56 (1.33, 1.84)*** | 2.17 (1.86, 2.53)*** | 2.96 (2.52, 3.48)*** |  |
| ≥ 60 | Ref | 1.61 (1.23, 2.11)*** | 2.15 (1.66, 2.79)*** | 2.64 (2.01, 3.47)*** |  |
| Sex |  |  |  |  | <0.001 |
| Male | Ref | 1.60 (1.43, 1.80)*** | 2.13 (1.91, 2.38)*** | 2.54 (2.26, 2.86)*** |  |
| Female | Ref | 1.84 (1.54, 2.19)*** | 2.94 (2.50, 3.47)*** | 4.45 (3.78, 5.25)*** |  |
| BMI |  |  |  |  | <0.001 |
| < 24 | Ref | 1.81 (1.51, 2.15)*** | 2.75 (2.33, 3.25)*** | 4.14 (3.50, 4.89)*** |  |
| ≥ 24 | Ref | 1.42 (1.28, 1.58)*** | 1.77 (1.60, 1.97)*** | 1.97 (1.76, 2.21)*** |  |
| Hypertension |  |  |  |  | <0.001 |
| No | Ref | 1.72 (1.52, 1.94)*** | 2.58 (2.30, 2.89)*** | 3.69 (3.29, 4.13)*** |  |
| Yes | Ref | 1.44 (1.21, 1.71)*** | 1.86 (1.57, 2.21)*** | 2.19 (1.81, 2.64)*** |  |
| Diabetes |  |  |  |  | 0.351 |
| No | Ref | 1.73 (1.55, 1.92)*** | 2.55 (2.31, 2.82)*** | 3.47 (3.13, 3.84)*** |  |
| Yes | Ref | 1.62 (1.13, 2.32)** | 1.84 (1.29, 2.63)** | 2.24 (1.57, 3.21)* |  |
| Dyslipidemia |  |  |  |  | 0.194 |
| No | Ref | 1.62 (1.44, 1.83)*** | 2.48 (2.22, 2.78)*** | 3.38 (3.03, 3.78)*** |  |
| Yes | Ref | 1.82 (1.56, 2.13)*** | 1.80 (1.54, 2.09)*** | 2.52 (2.17, 2.92)*** |  |

Adjusted for age, sex, smoking status, drinking status, hypertension, diabetes and dyslipidemia. In each subgroup, variables other than subgroup variables were adjusted

**P* < 0.05, ***P* < 0.01, and ****P* < 0.001

**Table S14.** Association between triglyceride-glucose × body mass index (TyG-BMI) and MASLD stratified by age, sex, BMI, hypertension, diabetes and dyslipidemia status

| Subgroup | Q1  *HR* (95%CI) | Q2  *HR* (95%CI) | Q3  *HR* (95%CI) | Q4  *HR* (95%CI) | *P* _interaction_ |
| --- | --- | --- | --- | --- | --- |
| Age |  |  |  |  | 0.061 |
| 18-44 | Ref | 2.73 (2.20, 3.38)*** | 6.85 (5.61, 8.36)*** | 13.98 (11.47, 17.04)*** |  |
| 45-59 | Ref | 2.45 (2.01, 2.99)*** | 4.14 (3.43, 5.01)*** | 7.26 (6.01, 8.77)*** |  |
| ≥ 60 | Ref | 3.02 (2.14, 4.25)*** | 4.32 (3.09, 6.03)*** | 7.41 (5.34, 10.28)*** |  |
| Sex |  |  |  |  | <0.001 |
| Male | Ref | 2.53 (2.22, 2.89)*** | 3.85 (3.39, 4.38)*** | 6.25 (5.49, 7.10)*** |  |
| Female | Ref | 3.11 (2.37, 4.07)*** | 7.79 (6.06, 10.02)*** | 19.11 (14.93, 24.45)*** |  |
| BMI |  |  |  |  | 0.001 |
| < 24 | Ref | 2.67 (2.12, 3.36)*** | 5.25 (4.23, 6.53)*** | 10.26 (8.28, 12.72)*** |  |
| ≥ 24 | Ref | 1.50 (1.35, 1.68)*** | 1.98 (1.78, 2.20)*** | 2.74 (2.45, 3.05)*** |  |
| Hypertension |  |  |  |  | 0.004 |
| No | Ref | 3.21 (2.70, 3.82)*** | 7.23 (6.14, 8.52)*** | 14.31 (12.17, 16.84)*** |  |
| Yes | Ref | 1.91 (1.55, 2.35)*** | 3.38 (2.78, 4.10)*** | 4.91 (4.05, 5.95)*** |  |
| Diabetes |  |  |  |  | 0.983 |
| No | Ref | 3.30 (2.84, 3.84)*** | 6.92 (6.00, 7.99)*** | 13.51 (11.72, 15.58)*** |  |
| Yes | Ref | 1.58 (1.06, 2.35)* | 2.41 (1.65, 3.51)*** | 3.48 (2.41, 5.03)** |  |
| Dyslipidemia |  |  |  |  | 0.550 |
| No | Ref | 3.16 (2.65, 3.77)*** | 7.10 (6.01, 8.38)*** | 14.40 (12.22, 16.97)*** |  |
| Yes | Ref | 2.48 (2.07, 2.97)*** | 3.49 (2.92, 4.16)*** | 5.14 (4.32, 6.13)*** |  |

Adjusted for age, sex, smoking status, drinking status, hypertension, diabetes and dyslipidemia. In each subgroup, variables other than subgroup variables were adjusted

**P* < 0.05, ***P* < 0.01, and ****P* < 0.001

**Table S15.** Association between triglyceride-glucose × waist circumference (TyG-WC) and MASLD stratified by age, sex, BMI, hypertension, diabetes and dyslipidemia status

| Subgroup | Q1  *HR* (95%CI) | Q2  *HR* (95%CI) | Q3  *HR* (95%CI) | Q4  *HR* (95%CI) | *P* _interaction_ |
| --- | --- | --- | --- | --- | --- |
| Age |  |  |  |  | 0.068 |
| 18-44 | Ref | 3.50 (2.83, 4.32)*** | 7.09 (5.80, 8.68)*** | 14.82 (12.09, 18.16)*** |  |
| 45-59 | Ref | 2.30 (1.90, 2.78)*** | 3.72 (3.09, 4.49)*** | 7.07 (5.84, 8.57)*** |  |
| ≥ 60 | Ref | 1.96 (1.44, 2.67)*** | 3.12 (2.34, 4.17)*** | 5.10 (3.81, 6.83)*** |  |
| Sex |  |  |  |  | 0.035 |
| Male | Ref | 2.15 (1.89, 2.44)*** | 3.36 (2.97, 3.79)*** | 5.46 (4.82, 6.18)*** |  |
| Female | Ref | 3.40 (2.64, 4.36)*** | 7.30 (5.76, 9.24)*** | 15.45 (12.22, 19.52)*** |  |
| BMI |  |  |  |  | <0.001 |
| < 24 | Ref | 3.12 (2.48, 3.94)*** | 6.12 (4.90, 7.63)*** | 10.88 (8.68, 13.64)*** |  |
| ≥ 24 | Ref | 1.43 (1.28, 1.60)*** | 1.97 (1.76, 2.20)*** | 2.73 (2.43, 3.07)*** |  |
| Hypertension |  |  |  |  | 0.041 |
| No | Ref | 3.60 (3.05, 4.24)*** | 6.93 (5.91, 8.12)*** | 14.13 (12.01, 16.61)*** |  |
| Yes | Ref | 1.98 (1.63, 2.41)*** | 2.98 (2.45, 3.62)*** | 4.68 (3.83, 5.71)*** |  |
| Diabetes |  |  |  |  | 0.740 |
| No | Ref | 3.43 (2.98, 3.94)*** | 6.38 (5.56, 7.31)*** | 12.65 (11.00, 14.55)*** |  |
| Yes | Ref | 1.96 (1.33, 2.89)** | 2.64 (1.81, 3.85)*** | 3.22 (2.20, 4.71)* |  |
| Dyslipidemia |  |  |  |  | 0.602 |
| No | Ref | 3.56 (3.02, 4.20)*** | 6.52 (5.55, 7.65)*** | 14.19 (12.07, 16.69)*** |  |
| Yes | Ref | 2.30 (1.93, 2.74)*** | 3.13 (2.62, 3.73)*** | 4.66 (3.89, 5.57)*** |  |

Adjusted for age, sex, smoking status, drinking status, hypertension, diabetes and dyslipidemia. In each subgroup, variables other than subgroup variables were adjusted

**P* < 0.05, ***P* < 0.01, and ****P* < 0.001

**Table S16.** Association between triglyceride-glucose × waist circumference/height (TyG-WHtR) and MASLD stratified by age, sex, BMI, hypertension, diabetes and dyslipidemia status

| Subgroup | Q1  *HR* (95%CI) | Q2  *HR* (95%CI) | Q3  *HR* (95%CI) | Q4  *HR* (95%CI) | *P* _interaction_ |
| --- | --- | --- | --- | --- | --- |
| Age |  |  |  |  | 0.615 |
| 18-44 | Ref | 2.71 (2.23, 3.30)*** | 5.90 (4.91, 7.09)*** | 10.41 (8.68, 12.50)*** |  |
| 45-59 | Ref | 2.16 (1.79, 2.60)*** | 3.47 (2.90, 4.15)*** | 6.21 (5.19, 7.43)*** |  |
| ≥ 60 | Ref | 1.92 (1.43, 2.57)*** | 2.61 (1.96, 3.47)*** | 4.05 (3.04, 5.39)*** |  |
| Sex |  |  |  |  | <0.001 |
| Male | Ref | 2.26 (1.99, 2.55)*** | 3.32 (2.95, 3.75)*** | 5.49 (4.84, 6.23)*** |  |
| Female | Ref | 2.89 (2.25, 3.71)*** | 7.53 (5.97, 9.49)*** | 15.08 (11.96, 19.01)*** |  |
| BMI |  |  |  |  | <0.001 |
| < 24 | Ref | 2.38 (1.93, 2.94)*** | 4.79 (3.94, 5.83)*** | 7.93 (6.49, 9.68)*** |  |
| ≥ 24 | Ref | 1.36 (1.22, 1.52)*** | 1.84 (1.66, 2.05)*** | 2.51 (2.25, 2.81)*** |  |
| Hypertension |  |  |  |  | 0.031 |
| No | Ref | 3.28 (2.80, 3.85)*** | 6.39 (5.48, 7.44)*** | 11.65 (10.00, 13.58)*** |  |
| Yes | Ref | 1.91 (1.58, 2.30)*** | 2.82 (2.34, 3.40)*** | 4.27 (3.53, 5.16)*** |  |
| Diabetes |  |  |  |  | 0.126 |
| No | Ref | 3.35 (2.92, 3.84)*** | 6.10 (5.34, 6.96)*** | 11.00 (9.62, 12.58)*** |  |
| Yes | Ref | 2.30 (1.54, 3.44)*** | 3.14 (2.12, 4.65)*** | 3.76 (2.52, 5.61)*** |  |
| Dyslipidemia |  |  |  |  | 0.238 |
| No | Ref | 3.07 (2.62, 3.60)*** | 6.23 (5.35, 7.25)*** | 11.48 (9.85, 13.38)*** |  |
| Yes | Ref | 2.07 (1.75, 2.45)*** | 2.81 (2.38, 3.31)*** | 4.09 (3.47, 4.83)*** |  |

Adjusted for age, sex, smoking status, drinking status, hypertension, diabetes and dyslipidemia. In each subgroup, variables other than subgroup variables were adjusted

**P* < 0.05, ***P* < 0.01, and ****P* < 0.001

**Table S17.** Associations between cardiometabolic indices and the incidence of MASLD after excluding individuals with MASLD occurring within 1 year of follow-up

| Variables | Model 1  *HR* (95%*CI*) | Model 2  *HR* (95%*CI*) | Model 3  *HR* (95%*CI*) |
| --- | --- | --- | --- |
| CMI |  |  |  |
| Q1 | Reference | Reference | Reference |
| Q2 | 2.35 (2.06, 2.67)*** | 2.17 (1.91, 2.48)*** | 2.16 (1.90, 2.46)*** |
| Q3 | 4.30 (3.81, 4.86)*** | 3.79 (3.35, 4.29)*** | 3.69 (3.26, 4.18)*** |
| Q4 | 7.94 (7.08, 8.92)*** | 6.42 (5.69, 7.24)*** | 5.68 (5.01, 6.44)*** |
| *P*_trend_ | <0.001 | <0.001 | <0.001 |
| AIP |  |  |  |
| Q1 | Reference | Reference | Reference |
| Q2 | 2.13 (1.89, 2.40)*** | 1.95 (1.73, 2.20)*** | 1.93 (1.71, 2.18)*** |
| Q3 | 3.52 (3.14, 3.94)*** | 3.02 (2.69, 3.39)*** | 2.93 (2.61, 3.28)*** |
| Q4 | 6.18 (5.55, 6.88)*** | 4.83 (4.32, 5.40)*** | 4.15 (3.69, 4.67)*** |
| *P*_trend_ | <0.001 | <0.001 | <0.001 |
| TyG |  |  |  |
| Q1 | Reference | Reference | Reference |
| Q2 | 1.92 (1.71, 2.14)*** | 1.78 (1.60, 2.00)*** | 1.74 (1.56, 1.95)*** |
| Q3 | 2.97 (2.67, 3.30)*** | 2.58 (2.32, 2.87)*** | 2.46 (2.21, 2.74)*** |
| Q4 | 4.89 (4.42, 5.40)*** | 3.98 (3.59, 4.42)*** | 3.31 (2.96, 3.70)*** |
| *P*_trend_ | <0.001 | <0.001 | <0.001 |
| TyG-BMI |  |  |  |
| Q1 | Reference | Reference | Reference |
| Q2 | 3.42 (2.92, 4.01)*** | 3.38 (2.89, 3.96)*** | 3.35 (2.86, 3.92)*** |
| Q3 | 7.58 (6.54, 8.79)*** | 7.14 (6.14, 8.31)*** | 6.88 (5.92, 8.01)*** |
| Q4 | 14.98 (12.97, 17.31)*** | 13.86 (11.94, 16.09)*** | 12.54 (10.78, 14.58)*** |
| *P*_trend_ | <0.001 | <0.001 | <0.001 |
| TyG-WC |  |  |  |
| Q1 | Reference | Reference | Reference |
| Q2 | 3.41 (2.95, 3.95)*** | 3.49 (3.02, 4.04)*** | 3.45 (2.98, 3.99)*** |
| Q3 | 6.37 (5.55, 7.32)*** | 6.56 (5.68, 7.58)*** | 6.23 (5.39, 7.20)*** |
| Q4 | 12.81 (11.20, 14.65)*** | 13.52 (11.69, 15.64)*** | 11.85 (10.21, 13.75)*** |
| *P*_trend_ | <0.001 | <0.001 | <0.001 |
| TyG-WHtR |  |  |  |
| Q1 | Reference | Reference | Reference |
| Q2 | 3.30 (2.87, 3.81)*** | 3.28 (2.84, 3.78)*** | 3.23 (2.80, 3.73)*** |
| Q3 | 6.11 (5.33, 7.00)*** | 6.02 (5.24, 6.91)*** | 5.77 (5.03, 6.63)*** |
| Q4 | 11.25 (9.86, 12.84)*** | 11.49 (10.00, 13.20)*** | 10.18 (8.83, 11.72)*** |
| *P*_trend_ | <0.001 | <0.001 | <0.001 |

Model 1: Unadjusted

Model 2: Adjusted for age and sex

Model 3: Adjusted for age, sex, smoking status, drinking status, hypertension, diabetes and dyslipidemia

HR hazard ratio, CI confidence interval, MASLD metabolic dysfunction-associated steatotic liver disease, CMI cardiometabolic index, AIP atherogenic index of plasma, TyG triglyceride-glucose index, TyG-BMI triglyceride-glucose × body mass index, TyG-WC triglyceride-glucose × waist circumference, TyG-WHtR triglyceride-glucose × waist circumference/height

**P* < 0.05, ***P* < 0.01, and ****P* < 0.001

**Table S18.** Associations between cardiometabolic indices and the incidence of MASLD after excluding individuals who used antihypertensive, hypoglycemic or lipid-lowing drugs

| Variables | Model 1  *HR* (95%*CI*) | Model 2  *HR* (95%*CI*) | Model 3  *HR* (95%*CI*) |
| --- | --- | --- | --- |
| CMI |  |  |  |
| Q1 | Reference | Reference | Reference |
| Q2 | 2.46 (2.17, 2.79)*** | 2.26 (1.99, 2.56)*** | 2.24 (1.98, 2.54)*** |
| Q3 | 4.61 (4.11, 5.19)*** | 3.99 (3.54, 4.49)*** | 3.90 (3.46, 4.39)*** |
| Q4 | 9.09 (8.13, 10.16)*** | 7.12 (6.34, 8.00)*** | 6.31 (5.60, 7.12)*** |
| *P*_trend_ | <0.001 | <0.001 | <0.001 |
| AIP |  |  |  |
| Q1 | Reference | Reference | Reference |
| Q2 | 2.21 (1.96, 2.48)*** | 2.01 (1.79, 2.26)*** | 1.99 (1.77, 2.24)*** |
| Q3 | 3.82 (3.42, 4.26)*** | 3.23 (2.89, 3.61)*** | 3.14 (2.81, 3.51)*** |
| Q4 | 7.04 (6.35, 7.82)*** | 5.39 (4.84, 6.00)*** | 4.63 (4.14, 5.18)*** |
| *P*_trend_ | <0.001 | <0.001 | <0.001 |
| TyG |  |  |  |
| Q1 | Reference | Reference | Reference |
| Q2 | 1.88 (1.69, 2.10)*** | 1.73 (1.56, 1.93)*** | 1.70 (1.53, 1.90)*** |
| Q3 | 3.08 (2.79, 3.41)*** | 2.63 (2.37, 2.91)*** | 2.52 (2.27, 2.79)*** |
| Q4 | 5.36 (4.87, 5.89)*** | 4.22 (3.82, 4.66)*** | 3.50 (3.15, 3.88)*** |
| *P*_trend_ | <0.001 | <0.001 | <0.001 |
| TyG-BMI |  |  |  |
| Q1 | Reference | Reference | Reference |
| Q2 | 3.43 (2.94, 3.99)*** | 3.32 (2.85, 3.87)*** | 3.29 (2.82, 3.83)*** |
| Q3 | 8.13 (7.04, 9.38)*** | 7.42 (6.41, 8.58)*** | 7.16 (6.18, 8.28)*** |
| Q4 | 17.24 (15.00, 19.82)*** | 15.12 (13.10, 17.46)*** | 13.71 (11.85, 15.86)*** |
| *P*_trend_ | <0.001 | <0.001 | <0.001 |
| TyG-WC |  |  |  |
| Q1 | Reference | Reference | Reference |
| Q2 | 3.52 (3.05, 4.06)*** | 3.55 (3.08, 4.10)*** | 3.51 (3.04, 4.05)*** |
| Q3 | 6.81 (5.95, 7.80)*** | 6.81 (5.92, 7.83)*** | 6.48 (5.63, 7.45)*** |
| Q4 | 14.99 (13.15, 17.08)*** | 14.96 (13.00, 17.22)*** | 13.09 (11.35, 15.11)*** |
| *P*_trend_ | <0.001 | <0.001 | <0.001 |
| TyG-WHtR |  |  |  |
| Q1 | Reference | Reference | Reference |
| Q2 | 3.56 (3.10, 4.10)*** | 3.48 (3.02, 4.00)*** | 3.43 (2.98, 3.95)*** |
| Q3 | 6.95 (6.08, 7.94)*** | 6.58 (5.74, 7.54)*** | 6.32 (5.51, 7.24)*** |
| Q4 | 13.35 (11.72, 15.20)*** | 12.64 (11.04, 14.48)*** | 11.18 (9.73, 12.83)*** |
| *P*_trend_ | <0.001 | <0.001 | <0.001 |

Model 1: Unadjusted

Model 2: Adjusted for age and sex

Model 3: Adjusted for age, sex, smoking status, drinking status, hypertension, diabetes and dyslipidemia

HR hazard ratio, CI confidence interval, MASLD metabolic dysfunction-associated steatotic liver disease, CMI cardiometabolic index, AIP atherogenic index of plasma, TyG triglyceride-glucose index, TyG-BMI triglyceride-glucose × body mass index, TyG-WC triglyceride-glucose × waist circumference, TyG-WHtR triglyceride-glucose × waist circumference/height

**P* < 0.05, ***P* < 0.01, and ****P* < 0.001

**Table S19.** Associations between cardiometabolic indices and the incidence of MASLD after grouping by optimal cutoff point

| Variables | Model 1  *HR* (95%*CI*) | Model 2  *HR* (95%*CI*) | Model 3  *HR* (95%*CI*) |
| --- | --- | --- | --- |
| CMI (n=14,550) |  |  |  |
| Low (≤ 0.4299) | Reference | Reference | Reference |
| High (> 0.4299) | 3.80 (3.58, 4.03)*** | 3.14 (2.95, 3.34)*** | 2.78 (2.60, 2.97)*** |
| AIP (n=12,828) |  |  |  |
| Low (≤ 0.0059) | Reference | Reference | Reference |
| High (> 0.0059) | 3.13 (2.96, 3.31)*** | 2.56 (2.42, 2.71)*** | 2.22 (2.09, 2.36)*** |
| TyG (n=14,064) |  |  |  |
| Low (≤ 8.6181) | Reference | Reference | Reference |
| High (> 8.6181) | 2.81 (2.66, 2.97)*** | 2.35 (2.22, 2.49)*** | 2.01 (1.89, 2.14)*** |
| TyG-BMI (n=13,214) |  |  |  |
| Low (≤ 206.3687) | Reference | Reference | Reference |
| High (> 206.3687) | 4.65 (4.39, 4.93)*** | 3.99 (3.75, 4.24)*** | 3.60 (3.38, 3.84)*** |
| TyG-WC (n=10,882) |  |  |  |
| Low (≤ 727.4760) | Reference | Reference | Reference |
| High (> 727.4760) | 4.26 (4.03, 4.51)*** | 3.70 (3.46, 3.95)*** | 3.25 (3.03, 3.47)*** |
| TyG-WHtR (n=12,936) |  |  |  |
| Low (≤ 4.1786) | Reference | Reference | Reference |
| High (> 4.1786) | 4.08 (3.85, 4.33)*** | 3.67 (3.44, 3.91)*** | 3.27 (3.06, 3.49)*** |

Model 1: Unadjusted

Model 2: Adjusted for age and sex

Model 3: Adjusted for age, sex, smoking status, drinking status, hypertension, diabetes and dyslipidemia

HR hazard ratio, CI confidence interval, MASLD metabolic dysfunction-associated steatotic liver disease, CMI cardiometabolic index, AIP atherogenic index of plasma, TyG triglyceride-glucose index, TyG-BMI triglyceride-glucose × body mass index, TyG-WC triglyceride-glucose × waist circumference, TyG-WHtR triglyceride-glucose × waist circumference/height

**P* < 0.05, ***P* < 0.01, and ****P* < 0.001

**Table S20.** Associations between cardiometabolic indices and the incidence of MASLD after propensity score matching

| Variables | *HR* (95%*CI*) |
| --- | --- |
| CMI (n=14,550) |  |
| Low (≤ 0.4299) | Reference |
| High (> 0.4299) | 2.46 (2.29, 2.64)*** |
| AIP (n=12,828) |  |
| Low (≤ 0.0059) | Reference |
| High (> 0.0059) | 1.60 (1.50, 1.72)*** |
| TyG (n=14,064) |  |
| Low (≤ 8.6181) | Reference |
| High (> 8.6181) | 1.60 (1.49, 1.71)*** |
| TyG-BMI (n=13,214) |  |
| Low (≤ 206.3687) | Reference |
| High (> 206.3687) | 2.88 (2.68, 3.09)*** |
| TyG-WC (n=10,882) |  |
| Low (≤ 727.4760) | Reference |
| High (> 727.4760) | 2.43 (2.26, 2.62)*** |
| TyG-WHtR (n=12,936) |  |
| Low (≤ 4.1786) | Reference |
| High (> 4.1786) | 2.67 (2.48, 2.87)*** |

The propensity score was obtained using a logistic regression model. The variables in the propensity score model for matching were included sex, age, smoking, drinking, hypertension, diabetes and dyslipidemia. The matching was constructed based on a 1:1 ratio using the nearest-neighbor matching with a caliper width of 0.01 without replacement. The balance of variables between the groups before and after matching was assessed using standardised mean difference (SMD), with a value of less than 0.10 indicating balance.

Adjusted for age, sex, smoking status, drinking status, hypertension, diabetes and dyslipidemia

HR hazard ratio, CI confidence interval, MASLD metabolic dysfunction-associated steatotic liver disease, CMI cardiometabolic index, AIP atherogenic index of plasma, TyG triglyceride-glucose index, TyG-BMI triglyceride-glucose × body mass index, TyG-WC triglyceride-glucose × waist circumference, TyG-WHtR triglyceride-glucose × waist circumference/height

**P* < 0.05, ***P* < 0.01, and ****P* < 0.001

**Table S21.** Analysis of the mediation between cardiometabolic indices and MASLD

|  | **Mediation effect (95%CI), P value** | |  |
| --- | --- | --- | --- |
|  | Indirect effect | Direct effect | Mediation |
| **BMI** |  |  |  |
| CMI | 0.777 (0.728, 0.830), <0.001 | 1.146 (1.059, 1.234), <0.001 | 40.40% |
| AIP | 1.088 (1.029, 1.147), <0.001 | 2.194 (2.054, 2.334), <0.001 | 33.16% |
| TyG | 0.511 (0.484, 0.540), <0.001 | 0.888 (0.821, 0.955), <0.001 | 36.51% |
| TyG-BMI | -0.003 (-0.006, -0.001), <0.001 | 0.035 (0.032, 0.038), <0.001 | -10.41% |
| TyG-WC | 0.003 (0.003, 0.004), <0.001 | 0.006 (0.005, 0.006), <0.001 | 37.36% |
| TyG-WHtR | 0.739 (0.678, 0.800), <0.001 | 0.786 (0.704, 0.867), <0.001 | 48.47% |
| **Neutrophils** |  |  |  |
| CMI | 0.068 (0.054, 0.085), <0.001 | 1.739 (1.653, 1.826), <0.001 | 3.77% |
| AIP | 0.102 (0.078, 0.126), <0.001 | 2.907 (2.773, 3.041), <0.001 | 3.38% |
| TyG | 0.053 (0.043, 0.064), <0.001 | 1.217 (1.153, 1.280), <0.001 | 4.14% |
| TyG-BMI | 0.001 (0.001, 0.001), <0.001 | 0.031 (0.030, 0.032), <0.001 | 1.89% |
| TyG-WC | 0.000 (0.000, 0.000), <0.001 | 0.009 (0.008, 0.009), <0.001 | 2.25% |
| TyG-WHtR | 0.039 (0.031, 0.049), <0.001 | 1.436 (1.377, 1.496), <0.001 | 2.66% |
| **Lymphocytes** |  |  |  |
| CMI | 0.075 (0.060, 0.088), <0.001 | 1.737 (1.650, 1.824), <0.001 | 4.12% |
| AIP | 0.111 (0.088, 0.134), <0.001 | 2.899 (2.766, 3.033), <0.001 | 3.69% |
| TyG | 0.052 (0.042, 0.061), <0.001 | 1.222 (1.158, 1.285), <0.001 | 4.05% |
| TyG-BMI | 0.001 (0.001, 0.001), <0.001 | 0.031 (0.030, 0.032), <0.001 | 2.50% |
| TyG-WC | 0.000 (0.000, 0.000), <0.001 | 0.009 (0.008, 0.009), <0.001 | 2.25% |
| TyG-WHtR | 0.042 (0.034, 0.049), <0.001 | 1.440 (1.380, 1.499), <0.001 | 2.81% |

Abbreviation: MASLD metabolic dysfunction-associated steatotic liver disease, CI confidence interval, BMI body mass index, CMI cardiometabolic index, AIP atherogenic index of plasma, TyG triglyceride-glucose index, TyG-BMI triglyceride-glucose × body mass index, TyG-WC triglyceride-glucose × waist circumference, TyG-WHtR triglyceride-glucose × waist circumference/height
